# Lipolysis pathways modulate lipid mediator release and endocannabinoid system signaling in dairy cows’ adipocytes

**DOI:** 10.1186/s40104-024-01062-z

**Published:** 2024-08-03

**Authors:** Madison N. Myers, Miguel Chirivi, Jeff C. Gandy, Joseph Tam, Maya Zachut, G. Andres Contreras

**Affiliations:** 1 Department of Large Animal Clinical Sciences, College of Veterinary Medicine, Michigan State University, East Lansing, MI 48824 USA; 2 Obesity and Metabolism Laboratory, The Institute for Drug Research, School of Pharmacy, Faculty of Medicine, The Hebrew University of Jerusalem, Jerusalem, 9112001 Israel; 3 Department of Ruminant Science, Institute of Animal Sciences, Agricultural Research Organization Volcani Institute, Rishon LeZion, 7505101 Israel

**Keywords:** Adipose tissue, Dairy cows, Endocannabinoid system, Endocannabinoids, Lipolysis

## Abstract

**Background:**

As cows transition from pregnancy to lactation, free fatty acids (FFA) are mobilized from adipose tissues (AT) through lipolysis to counter energy deficits. In clinically healthy cows, lipolysis intensity is reduced throughout lactation; however, if FFA release exceeds tissue demands or the liver’s metabolic capacity, lipid byproducts accumulate, increasing cows’ risk of metabolic and infectious disease. Endocannabinoids (eCBs) and their congeners, *N-*acylethanolamines (NAEs), are lipid-based compounds that modulate metabolism and inflammation. Their synthesis and release depend upon the availability of FFA precursors and the abundance of synthesizing and degrading enzymes and transporters. Therefore, we hypothesized that eCB production and transcription of endocannabinoid system components are modulated by lipolysis pathways in adipocytes. To test this hypothesis, we stimulated canonical (isoproterenol, 1 µmol/L; ISO) and inflammatory (lipopolysaccharide, 1 µg/mL; LPS) lipolysis pathways in adipocytes isolated from the AT of 5 Holstein dairy cows. Following, we assessed lipolysis intensity, adipocytes’ release of eCBs, and transcription of endocannabinoid system components.

**Results:**

We found that ISO and LPS stimulated lipolysis at comparable intensities. Exposure to either treatment tended to elevate the release of eCBs and NAEs by cultured adipocytes; however, specific eCBs and NAEs and the transcriptional profiles differed by treatment. On one hand, ISO enhanced adipocytes’ release of 2-arachidonoylglycerol (2-AG) but reduced NAE production. Notably, ISO enhanced the cells’ expression of enzymes associated with 2-AG biosynthesis (*INPP5F*, *GDPD5*, *GPAT4*), transport (*CD36*), and adipogenesis (*PPARG*). Conversely, LPS enhanced adipocytes’ synthesis and release of *N-*arachidonoylethanolamide (AEA). This change coincided with enhanced transcription of the NAE-biosynthesizing enzyme, *PTPN22*, and adipocytes’ transcription of genes related to eCB degradation (*PTGS2*, *MGLL*, *CYP27B1*). Furthermore, LPS enhanced adipocytes’ transcription of eCB and NAE transporters (*HSPA1A*, *SCP2*) and the expression of the anti-adipogenic ion channel, *TRPV3*.

**Conclusions:**

Our data provide evidence for distinct modulatory roles of canonical and inflammatory lipolysis pathways over eCB release and transcriptional regulation of biosynthesis, degradation, transport, and ECS signaling in cows’ adipocytes. Based on our findings, we conclude that, within adipocytes, eCB production and ECS component expression are, at least in part, mediated by lipolysis in a pathway-dependent manner. These findings contribute to a deeper understanding of the molecular mechanisms underlying metabolic regulation in dairy cows’ AT, with potential implications for prevention and treatment of inflammatory and metabolic disorders.

**Graphical Abstract:**

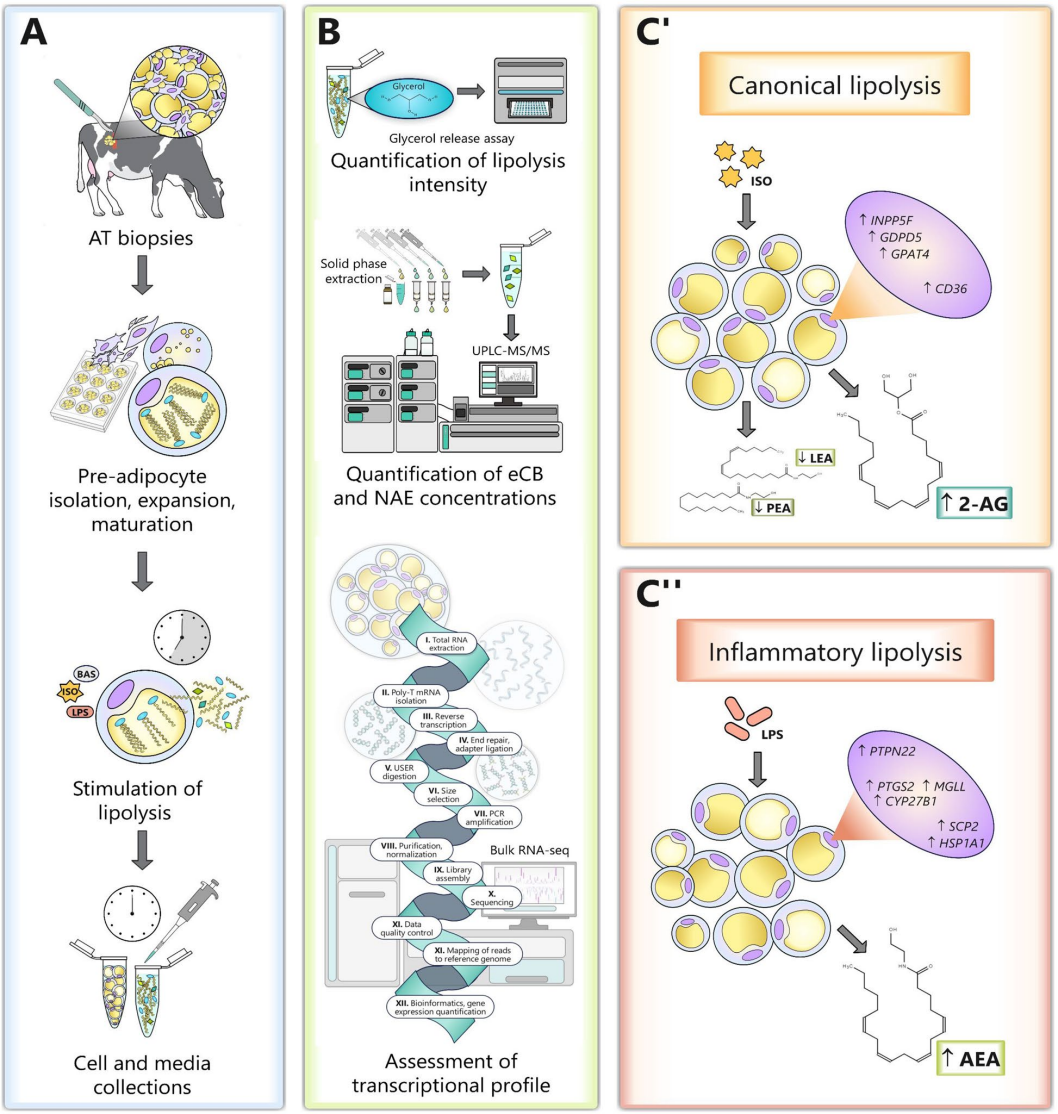

**Supplementary Information:**

The online version contains supplementary material available at 10.1186/s40104-024-01062-z.

## Background

The susceptibility of dairy cows to metabolic and infectious disease is greatest during the periparturient period (PP); a physiological stage defined as the 3 weeks preceding and 3 weeks following calving [1]. To meet nutrient demands associated with lactogenesis and calving, extensive metabolic and immunologic adaptations occur—especially in cows’ adipose tissues (AT) [2]. During this time, free fatty acids (FFA) are rapidly mobilized from adipocytes within AT through lipolysis, buffering energy availability according to physiologic demands [3]. This mechanism relies upon the activation of three key lipases–adipose triglyceride lipase (ATGL), hormone-sensitive lipase (HSL), and monoacylglycerol lipase (MGLL)—which sequentially break down stored lipids, releasing FFA and glycerol from AT [4]. The primary pathway, known as canonical lipolysis, is initiated upon the stimulation of growth hormone or β-adrenergic receptors (β-AR), among others, by their respective ligands [3]. Adenylyl cyclase activation triggers the phosphorylation of protein kinase A (PKA), which stimulates perilipin 1 (PLIN1) and HSL [4]. The activation of ATGL is dependent upon binding of its cofactor, α/β hydrolase domain-containing protein 5 (ABHD5), which is freed from PLIN1 upon its phosphorylation, initiating triacylglycerol hydrolysis and FFA release [5, 6].

While essential short-term, intense lipolysis that persists into lactation may result in the accumulation of toxic lipid byproducts within AT and other tissues [7, 8]. Known as lipolysis dysregulation, this process impairs local and systemic immune responses, increasing the susceptibility of near-calving cows to costly metabolic and inflammatory diseases, such as metritis, ruminal acidosis, and mastitis [8, 9]. These conditions favor the invasion of pathogenic bacteria and accumulation of bacterial fragments such as lipopolysaccharide (LPS) in the blood and peripheral tissues, which may lead to clinical endotoxemia [10]. Recent evidence published by our group demonstrates that, in bovine adipocytes, toll-like receptor-4 (TLR-4) activation by LPS triggers lipolysis through an alternative, extracellular signal-regulated kinase-1/2 (ERK1/2)-mediated cascade. Through this process, known as inflammatory lipolysis, in addition to PKA-mediated stimulation of the canonical pathway, LPS drives lipid mobilization from adipocytes [11, 12].

The endocannabinoid system (ECS) is a complex signaling network consisting of specialized receptors, fatty acid-derived ligands, and enzymes responsible for their synthesis, degradation, and transport [13]. The ECS is recognized as a potent mediator of metabolic and inflammatory processes in mammals [13, 14]. Such effects result from the stimulation of diverse receptor families found within the ECS, which are widely distributed throughout the body, yet differentially expressed between cell types and tissues. For example, the metabolically active cannabinoid-1 receptor (CB1R), a G protein-coupled receptor (GPR), is highly expressed throughout nervous tissues and the surfaces of peripheral cells, including adipocytes. In the adipocytes of dairy cows, specifically, activation of CB1R enhances lipid storage and reduces FFA mobilization [15, 16]. In contrast, the cannabinoid-2 receptor (CB2R) modulates inflammatory responses and is predominantly expressed on the surface of immune cells [15, 17]. In cows exhibiting greater PP lipolysis, AT expression of *CNR1* and *CNR2* (corresponding to CB1R and CB2R, respectively) are enhanced, although the direct role lipolysis plays in the regulation of ECS-associated gene transcription is, to the authors’ knowledge, yet to be revealed [18]. Additional ECS receptors include GPR (GPR18, -55, -119) [19, 20], ion channels such as transient potential vanilloid channels-1 and -3 (TRPV1, -3) [21] and voltage-dependent calcium channels [22], and the transcription factor peroxisome proliferator-activated receptor gamma (PPARγ) [23, 24].

The stimulation of ECS receptors is dependent upon the availability and specificity of their ligands, endocannabinoids (eCBs) and structurally similar compounds, *N*-acylethanolamines (NAEs). Molecules in the former class, eCBs, include the two well-known arachidonic acid (AA; C20:4, n-6)-based compounds, 2-arachidonoylglycerol (2-AG) and *N-*arachidonoylethanolamide (anandamide, AEA; C20:4 NAE). These compounds, in contrast to non-eCBs (e.g., NAEs), bind and interact with CB1R and CB2R in addition to other ECS receptors, ion channels, and transcription factors [25]. The latter, NAEs, are named according to the identity of their FFA acyl chain donor, and include AEA, linoleoylethanolamide (LEA; C18:2 NAE), oleoylethanolamide (OEA; C18:1 NAE), palmitoylethanolamide (PEA; C16:0 NAE), and stearoylethanolamide (SEA; C18:0 NAE), along with others (extensively reviewed in [26]). These molecules, along with eCBs, exert local and systemic anti-inflammatory, orexigenic, and anti-nociceptive effects through their binding and activation of ECS receptors, which may be of benefit to PP cows, especially [27].

Several factors, including cellular stress or injury, inflammation, and changes in metabolic status (e.g., fed versus fasting conditions), may stimulate the production of eCBs and NAEs [28]. Initially, biosynthesis of the compounds begins with the cleavage of FFA from an intracellular lipid source by lipases (DAGLα/β), phospholipases (NAPE-PLD, PLC), lysophospholipases (ABHD4), *N*-acyltransferases (NAT), or phospholipid phosphatases (PLPP). In non-adipocytes, the plasma membrane is considered the primary site of eCB synthesis and its resident phospholipids (PL), the primary sources of precursor FFA [29, 30]. In adipocytes, however, wherein hydrolysis of TAG by neutral lipases (ATGL, HSL) and FFA mobilization from the lipid droplet are near-constant, the effects of lipolysis on eCB biosynthesis are yet to be determined. Considering that lipolysis and inflammation are known modulators of intra- and extracellular FFA abundance and membrane PL composition, we anticipate that the biosynthesis and release of eCBs and NAEs are altered upon the stimulation of AT lipolysis pathways in the adipocytes of PP cattle [31, 32]. Such notions are supported by the previous work of Zachut et al., which revealed that greater body fat mobilization (e.g., lipolysis) post-calving is associated with elevations in AT AA, 2-AG, and AEA concentrations [18]. In the same study, PEA and OEA levels were also enhanced in AT and plasma from pre- to postpartum, corresponding with transient shifts in availability of the respective precursor FFA (palmitic, C16:0; oleic, C18:1) released from the AT of PP cows [8].

Both eCBs and NAEs are susceptible to rapid breakdown by hydrolyzing and oxidizing enzymes present in AT [13]. Hydrolysis of 2-AG is performed by ABHD6, -12, and MGLL, whereas fatty acid amide hydrolase (FAAH) and *N*-acylethanolamine acid amidase hydrolyze NAEs. Many oxidative enzymes upregulated during inflammation and intense lipolysis, including cyclooxygenases (COX), lipoxygenases (LOX), and cytochrome P450 monooxygenases (CYP), degrade the FFA-based ligands and may regulate their concentrations in bovine AT [3, 8, 33]. Taken together, byproducts of eCB and NAE degradation may reciprocally modulate lipolysis intensity and inflammation in the AT of PP cows, although much remains unknown in this context [8, 13].

**Fig. 1:**
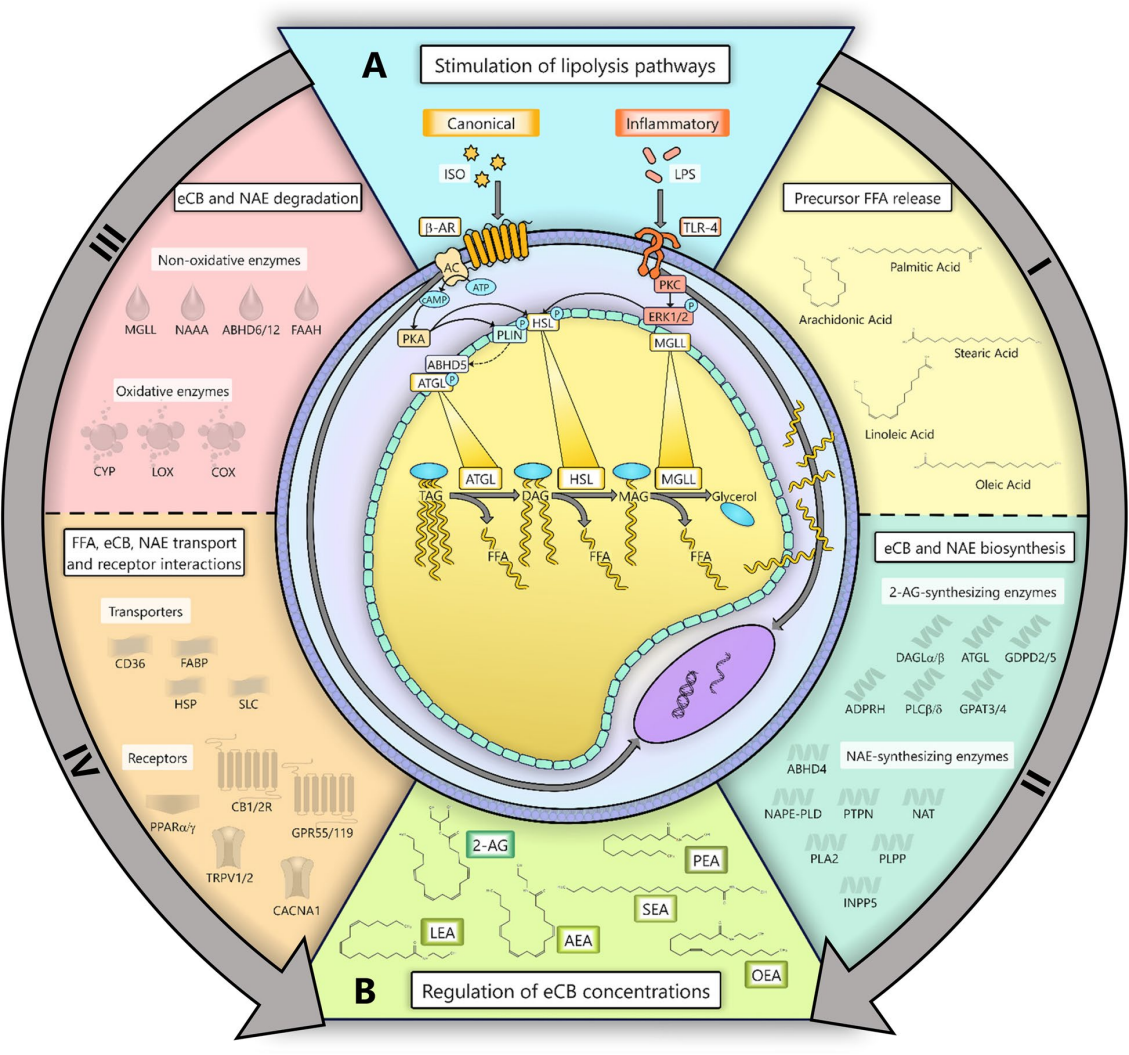
Hypothesized mechanisms by which lipolysis pathways regulate endocannabinoid (eCB) and *N*-acylethanolamine (NAE) concentrations in bovine adipose tissues (AT). **A** Outline of key signaling pathways involved in canonical and inflammatory lipolysis activation in adipocytes which may: (I) Enhance the availability of free fatty acid (FFA) precursors of eCBs and NAEs and alter their profile in adipocytes. (II) Modulate eCB synthesis by modifying the transcription of key eCB- and NAE-synthesizing gene networks. (III) Promote the degradation of eCBs and NAEs by enhancing the transcription of oxidizing and hydrolyzing enzymes. (IV) Regulate the release and activity of eCBs and NAEs by altering the expression and abundance of eCBs and NAE transporters and endocannabinoid system (ECS)-associated receptors. **B** Key FFA-derived eCBs and their congeners, NAEs, within in the AT ECS

While greater body fat mobilization (e.g., lipolysis) and inflammation post-calving are associated with elevated eCB concentrations in cows’ AT, plasma, and endometrial tissues, the direct implications of canonical and inflammatory lipolysis pathway activation on eCB production, degradation, and signaling in AT remain unknown [14, 18]. We hypothesized that stimulation of lipolysis pathways enhances the biosynthesis and release of eCBs and NAEs in bovine adipocytes by increasing FFA availability and altering the transcriptional profile of ECS-associated gene networks (Fig. 1). Using an in vitro adipocyte culture model, we tested this hypothesis by determining the effects of canonical and inflammatory lipolysis pathway stimulation on eCB release and transcriptional changes in eCB- and NAE-synthesizing, degrading, transporting, and receptor gene networks.

## Methods

### Animals and tissue collections

All procedures were approved by the Institutional Animal Care and Use Committee at Michigan State University (AUF #11-16-188-00) and performed in accordance with local and federal guidelines.

Five healthy, multiparous, non-gestating, non-lactating Holstein cows from commercial Michigan dairy farms were selected from a local abattoir. Before slaughter, animals were assigned a body condition score (BCS) by a trained veterinarian. To limit confounding variables, the BCS of all selected animals fell within the range of 3.25 to 3.75. Following euthanasia by captive bolt and jugular exsanguination, internal organs of candidate animals were evaluated. Animals with evidence of intra-abdominal, thoracic, or gastrointestinal disease were excluded. Following hide removal, 5 g of subcutaneous AT was collected from the right paralumbar fossa (flank) region. AT samples were immediately added to 30 mL filtered Krebs–Ringer bicarbonate buffer (KRBB, pH 7.4) formulated as previously described [16]. Samples were transported at 37 °C.

### Adipocyte progenitor isolation and adipocyte culture

Upon arrival at the laboratory, ~ 50 mg of each AT sample was finely minced using sterile surgical scissors into 1–2 mm^3^ fragments. Tissue pieces were transferred into a 12-well culture plate (four fragments per well) and spaced approximately 5 mm apart. After 10 min of incubation at 37 °C, 300 µL of MesenPro RS Complete Medium (Thermo Fisher Scientific, Waltham, MA, USA) supplemented with 1% L-glutamine (Gibco, Waltham, MA, USA) and 0.25 µg/mL amphotericin B (Antibiotic/Antimycotic solution; Gibco) was carefully added to each well and plates were incubated at 37 °C. Medium was changed every 48 h until preadipocytes migrated onto the plate surface from AT fragments and reached confluency. Following, cells were lifted by trypsinization (Gibco) and passed into flasks containing preadipocyte medium consisting of 10% fetal bovine serum (FBS; Corning), Dulbecco's modified Eagle medium/F12 (Corning, NY, USA), 44.05 mmol/L sodium bicarbonate (Corning), 100 µmol/L ascorbic acid (Sigma-Aldrich, St. Louis, MO, USA), 33 µmol/L biotin (Sigma-Aldrich), 17 µmol/L pantothenate (Sigma-Aldrich), 20 mmol/L HEPES (Teknova, Hollister, CA, USA), 1% L-glutamine (Gibco), and supplemented with 100 U/mL penicillin, 100 µg/mL streptomycin, 0.25 µg/mL amphotericin B (Antibiotic/Antimycotic Solution; Gibco).

Following expansion (2 to 3 serial passages), preadipocytes were seeded at a density of 20,000 cells/cm^2^ in 6-well plates [34]. Once confluency was reached, preadipocyte medium was removed and replaced with adipocyte induction medium: pre-adipocyte medium supplemented with 10 mg/mL insulin (Sigma-Aldrich), 1 µmol/L octanoic acid (Acros Organics, Geel, Belgium), 10 mmol/L acetate (Sigma-Aldrich), 10 µg/mL transferrin (Sigma-Aldrich), 5 µmol/L troglitazone (AdipoGen Life Sciences, San Diego, CA, USA) supplemented with 1 µmol/L 3-isobutyl-1-methylxanthine (IBMX; AdipoGen), and 0.5 µmol/L dexamethasone (Cayman Chemical, Ann Arbor, MI, USA). Following 48 h of incubation, 60% of the induction medium volume was removed and replaced with an equivalent volume of adipocyte medium (preadipocyte medium with 10 mg/mL insulin, 1 µmol/L octanoic acid, 10 mmol/L acetate, 10 µg/mL transferrin, and 5 µmol/L troglitazone). Cells were incubated at 37 °C and medias were refreshed every 48 h for 7 d.

### Lipolysis assays

After 7 d of induction, mature adipocytes were incubated in serum-free culture medium consisting of KRBB + 2% BSA (Sigma-Aldrich) only (basal lipolysis; BAS) or with 1 µmol/L isoproterenol (ISO) or 1 µg/mL lipopolysaccharide (LPS). After 7 h of incubation, cells and media were collected according to assay specifications detailed below, snap-frozen in liquid nitrogen, and stored at –80 °C until further analysis.

### Quantification of lipolysis

Lipolytic responses of adipocytes were assessed using the Glycerol-Glo kit (Promega Corp., Madison, WI, USA). Briefly, 50 µL of treated sample medium was added to a 96-well plate, followed by 1 h of incubation with assay reagents according to the manufacturer’s guidelines. Glycerol release was normalized by protein content for each sample following extraction in RIPA buffer (Teknova) supplemented with protease and phosphatase inhibitor cocktail (Thermo Fisher Scientific), as determined by Pierce™ BCA reagent quantification [35].

### Endocannabinoid quantification

Solid phase extraction began with 1:1 dilution of media samples (1 mL) in HPLC-grade water (1 mL). Next, 20 µL of methanol containing 10 ng of each internal eCB and NAE standard (arachidonoyl ethanolamide-d_8_, 1-arachidonoyl-d_5_-rac-glycerol, 1-arachidonoyl-d_8_-rac-glycerol, and 2-arachidonoyl-glycerol-d_8_, palmitoyl ethanolamide-d_5_, oleoyl ethanolamide-d_4_, stearoyl ethanolamide-d_3_, linoleoyl ethanolamide-d_4_; 0.5 µg/mL each in methanol; Cayman Chemical) was added to each diluted sample. Following thorough mixing, samples were sonicated for 2 min, then carefully transferred to Oasis^®^ PRiME HLB solid phase extraction cartridges (3 mL, 150 mg Sorbent; Waters Corporation, Milford, MA, USA) and allowed to drip through for 5 min. Next, cartridges were eluted twice with 1 mL of hexane–ethyl acetate (8:2; Sigma-Aldrich) and evaporated in a SpeedVac (Thermo Fisher Scientific). After, cartridges were eluted with 100 µL of methanol, and extract suspensions were thoroughly vortexed. Then, 20 μL water containing LC/MS-grade ammonium formate 10 mmol/L (6.3 mg/mL water; Sigma-Aldrich) and 1% formic acid (10 µL/mL water; Sigma-Aldrich) was added to each sample.

Quantification of eCBs and NAEs was achieved using the methods described by Ney et al. [36] with some modifications. Ultra performance liquid chromatographic (UPLC) separation was performed with the Waters ACQUITY^®^ H-class UPLC system (Waters Corp.). ACQUITY UPLC™ Bridged Ethyl Hybrid C18 columns (2.1 mm × 100 mm × 1.7 μm; Waters Corp.) were used. The UPLC instrument was operated with mobile phase A consisting of 2.0 mmol/L ammonium acetate adjusted to pH 4.5 (Solvent A), and acetonitrile (Solvent B). For chromatographic separation, a gradient was applied. First, 30% B was held for 30 s, gradually increased over the next 30 s to 50%, then 70% over 3 min. The final gradient was applied over 2 min to reach 90% B, at which conditions samples were held for 2 min and 30 s. Following, samples were returned to initial conditions and re-equilibrated for 3 min. A flow rate of 0.3 mL/min was used and columns were held at 50 °C.

The UPLC system was coupled to a Waters Xevo^®^ TQ-XS triple quadrupole mass spectrometer (Waters Corp.) with multiple reaction monitoring (MRM) used for analysis. The instrument was operated in positive electrosonic spray ionization mode with a capillary voltage of 2.7 kV, and, for each MRM stage, collision energies and cone voltages were optimized. A capillary temperature of 450 °C was maintained for desolvation. Pure nitrogen gas was injected at a rate of 950 L/h for nebulization and, within the cone, 50 L/h.

### RNA extraction

Adipocytes were rinsed twice with 1 × phosphatase buffered saline (PBS; Teknova) and lifted by trypsinization. Suspensions were transferred to 0.5 mL microcentrifuge tubes and cells were pelleted at 300 × *g* for 3 min. Following, supernatants were removed by pipetting. Cells were resuspended in 200 μL of cold 1-thioglycerol/homogenization solution (Promega Corp.), flash frozen in liquid nitrogen, and samples were stored at –80 °C.

Prior to extraction, samples were thawed on ice. RNA was extracted and purified using the automated Maxwell^®^ RSC Instrument (Promega Corp.) and simplyRNA Cells Kit (Promega Corp.) according to the manufacturer’s instructions. Samples were stored at –80 °C until further processing. Following, RNA purity and concentration were determined using the NanoDrop 1000 Spectrophotometer (Thermo Fisher Scientific) and an RNA integrity number (RIN) was assigned to each sample using the 4200 TapeStation automated electrophoresis instrument (Agilent, Santa Clara, CA, USA) at the Michigan State University Genomics Core (East Lansing, MI, USA). All samples met quantity and purity requirements for transcriptomic sequencing; A_260_/A_230_ ≥ 2.0, RIN ≥ 9.5 (Novogene Corporation, Beijing, China).

### Bulk RNA-seq analysis

A subset of BAS-, ISO-, and LPS-treated samples from 3 animals were randomly selected for next-generation sequencing (NGS) of sample bulk RNA (RNA-seq). Samples were then shipped to Novogene Corporation (Sacramento, CA, USA) for RNA-seq reporting. Upon arrival, quality control analyses were performed for all samples. Next, poly-T oligo-attached magnetic beads were used to purify messenger RNA from 5 µg total RNA. The resulting transcripts were fragmented, and random hexamer primers were used to reverse transcribe the first strand of cDNA. For the second strand, cDNA was synthesized using dTTP (for non-directional, paired-end library construction) [37]. Following end repair, A-tailing, adapter ligation, size selection, amplification, and purification, comparative libraries were constructed. Library quality controls were certified after quantification with the Qubit^®^ RNA Broad-Range Assay (Thermo Fisher) and real-time PCR, and for detection of size distribution, an Agilent Bioanalyzer 2100 system (Agilent Technologies, Santa Clara, CA, USA). Quantified libraries were pooled, and samples were sequenced on the Hiseq PE150 platform based on the effective library concentration, data amount, and according to the manufacturer’s instructions (Illumina, San Diego, CA, USA). Sequencing depths ranged from 29 to 59 million paired-end 100 bp reads per sample (Additional file 1 [38]). Raw reads in FASTQ format were processed through in-house Perl scripts. Clean reads were obtained following the removal of adapter-containing, ploy-N, and low-quality reads. During this step, quality scores of 20 (Q20), 30 (Q30), and guanine-cytosine (GC)-content were simultaneously evaluated. Through Hisat2 (v.2.0.5), paired-end clean reads were mapped to the cow genome, ARS-UCD1.2 (https://www.ebi.ac.uk/ena/data/view/GCA_002263795.2) [39]. Mapped reads were assembled using StringTie (v.1.3.3b) [40]. The featureCounts program (v1.5.0-p3) was used to count mapped read numbers that were used for gene expression analysis [41]. All data is available in the NCBI Gene Expression Omnibus under accession number GSE267141.

### Statistical analyses

#### Assessment of metabolomics data

Statistical analyses were performed in JMP^®^ (v.14, SAS Institute Incorporated, Cary, NC, USA). The normality of variables was calculated using the Shapiro–Wilk test (*P* < 0.05). When non-normal, raw values were log_10_-transformed. Transformed variables included AEA, 2-AG, LEA, and OEA concentrations. Following transformation, all data met normality assumptions required for statistical analysis. One-way ANOVA pairwise comparisons with Tukey’s HSD were calculated and used to identify differences in glycerol, eCB, and NAE concentrations among treatment groups. For ease of interpretation, non-transformed values are presented in all related figures.

#### RNA-seq data analysis

Differential gene expression analyses were performed based on negative binomial distributions using the DESeq2R package (v1.20.0, DESeq2R development team) [42, 43]. DESeq2-normalized gene counts were log_2_-transformed, Student’s *t*-tests were conducted, and Benjamini and Hochberg’s approach for controlling false discovery rate (FDR) was used to adjust *P*-values [44]. Genes were considered differentially expressed (DEGs) when the log_2_ of the fold-change exceeded 0.58 (fold-change value > 1.5) and the −log_10_ of the adjusted *P*-value was above 1.30103 (*P* ≤ 0.05).

Gene Ontology enrichment analyses were performed using the online graphical gene-set enrichment tool, ShinyGO (v0.80, ShinyGO development team) [45], for functional clustering of DEGs. Significance was set to *P* < 0.05 for the interfacing Kyoto Encyclopedia of Genes and Genomes (KEGG) [46] analysis (Additional file 2 [47]).

To visualize differences in DEGs among comparison groups, volcano plots were created in GraphPad Prism (v.10 for Windows, GraphPad Software, Boston, MA, USA). Of all assessed, 183 ECS-affiliated genes were selected and grouped by function (Additional file 3 [48]). One-way ANOVA with Fisher’s LSD were then performed using DESeq2-normalized counts.

## Results

### Canonical and inflammatory lipolysis pathways differentially alter endocannabinoid biosynthesis and release by adipocytes

**Fig. 2:**
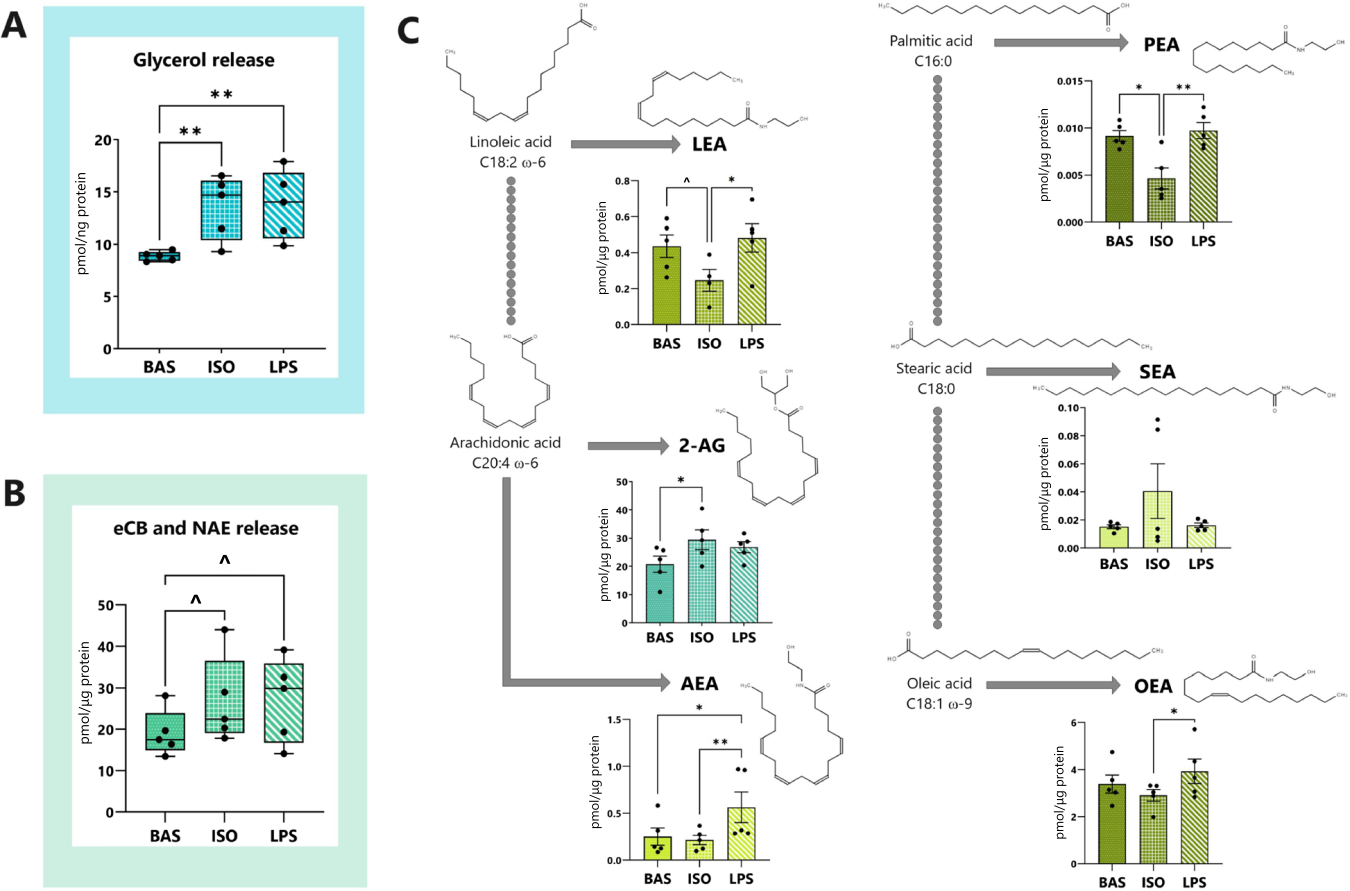
Lipolysis intensity and eCB abundance are modulated by canonical and inflammatory lipolysis pathways in dairy cows’ adipocytes. **A** Glycerol release (pmol/ng protein) by bovine adipocytes following 7 h of canonical (ISO, 1 µmol/L), and inflammatory (LPS, 1 µg/mL), and basal (BAS) lipolysis. **B** Total abundance of the endocannabinoids (eCBs), 2-arachidonylglycerol (2-AG) and *N-*arachidonoylethanolamide (AEA), and the *N*-acylethanolamines (NAEs), linolenoyl ethanolamide (LEA), oleoyl ethanolamide (OEA), stearoyl ethanolamide (SEA), and palmitoyl ethanolamide (PEA), released by adipocytes into the media following lipolytic challenge. Values reported in pmol/µg protein. **C** The influence of canonical and inflammatory lipolysis pathway activation on the release of 2-AG, AEA, LEA, OEA, PEA, and SEA by adipocytes into media. 2-AG is represented in teal and NAEs are shown in green. Values reported in pmol/µg protein. Asterisks indicate differences between groups (*P* < 0.1^, < 0.05*, < 0.01**) as determined by ANOVA and Tukey’s HSD; each dot represents an individual datapoint and error bars, SEM. *n* = 5

Cultured adipocytes were exposed to activators of canonical (ISO) and inflammatory (LPS) lipolysis pathways for 7 h. Following, lipolysis intensity was assessed through the quantification of glycerol release by adipocytes (Fig. 2A). The lipolytic responses of ISO- and LPS-treated adipocytes exceeded BAS levels by 1.5- and 1.6-fold, respectively, but were not different between lipolytic agents.

We then assessed the effects of targeted lipolysis pathway stimulation on the general trends in combined eCB and NAE biosynthesis and release by adipocytes (Fig. 2B). Relative to BAS, exposure to ISO (*P* < 0.09) or LPS (*P* < 0.08) tended to enhance adipocytes’ overall release of the 6 eCBs and NAEs quantified.

Next, we evaluated the impacts of canonical and inflammatory lipolysis pathway activation on adipocytes’ production of individual eCB and NAE (Fig. 2C). Relative to BAS, ISO enhanced the release of AA-derived 2-AG (29.4 ± 3.5 in ISO vs. 20.1 ± 2.9 in BAS; *P* < 0.05) but not AEA (0.22 ± 0.05 in ISO vs. 0.25 ± 0.09 in BAS; *P* > 0.1). AEA release was elevated in LPS-containing adipocyte media (0.56 ± 0.16) beyond BAS (*P* < 0.05) and ISO (*P* < 0.01) levels. Relative to BAS, ISO stifled adipocytes’ secretion of PEA (0.0046 ± 0.0016 in ISO vs. 0.0092 ± 0.0006 in BAS; *P* < 0.05) and tended to reduce LEA concentrations (0.25 ± 0.05 in ISO vs. 0.44 ± 0.06 in BAS; *P* < 0.1). ISO-treated adipocytes released less LEA (*P* < 0.05), OEA (3.39 ± 0.38; *P* < 0.05), and PEA (*P* < 0.01) relative to cells treated with LPS (0.48 ± 0.27 for LEA, 2.91 ± 0.25 for OEA, and 0.48 ± 0.27 for PEA in LPS). No differences were detected in SEA concentrations among treatment groups (*P* > 0.1).

### Adipocytes’ transcriptomic profiles are modulated by canonical and inflammatory lipolysis pathways

**Fig. 3:**
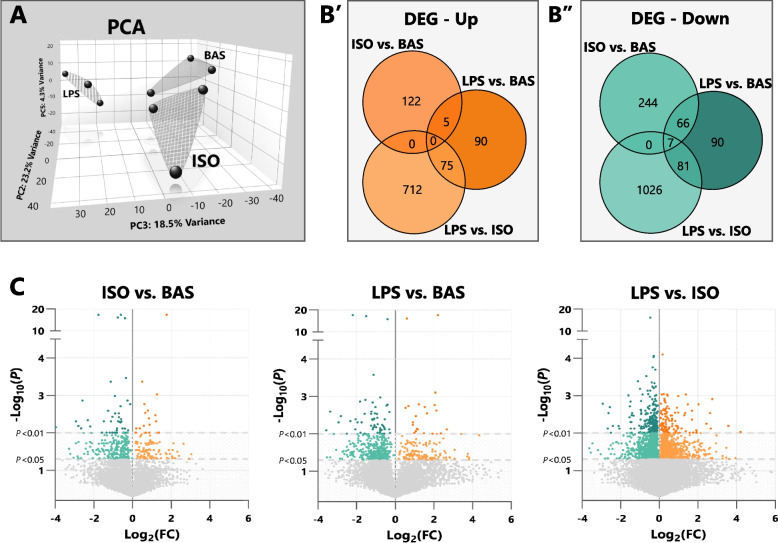
Canonical and inflammatory lipolysis pathways differentially alter the transcriptomic profiles of bovine adipocytes. Bovine adipocytes were exposed to isoproterenol (ISO; 1 µmol/L), lipopolysaccharide (LPS; 1 µg/mL), or untreated (BAS) for 7 h. Following, bulk RNA-seq analyses were performed (*n* = 3). **A** Principal component analysis (PCA) plot depicting sample clusters exhibiting similar gene expression patterns among treated adipocyte groups. **B** Venn diagrams depicting numbers of differentially expressed genes (DEG) and corresponding directional changes (B′, upregulated; B′′, downregulated) among comparison groups. **C** Volcano plots of differentially expressed genes among adipocyte samples

To assess transcriptional differences among BAS, ISO, and LPS, NGS bulk RNA-seq analyses were performed. PCA revealed clear separations between sample clusters among treatment groups (Fig. 3A). PC2 accounted for 23.1% of sample variance, followed by PC3 at 18.5%, and PC5 at 4.3%. The greatest transcriptional differences were observed in LPS vs. ISO (1,901 DEGs), followed by LPS vs. BAS (619 DEG), and ISO vs. BAS (444 DEG) (Fig. 3B and C). Relative to ISO, exposure to LPS upregulated the expression of 787 genes and downregulated that of 1,114. Transcription of 170 genes was upregulated and 244, downregulated, in LPS compared to BAS. Compared to BAS, ISO enhanced mRNA expression of 127 genes and reduced that of 317.

### Lipolysis pathways differentially modulate expression of eCB-synthesizing gene networks in bovine adipocytes

**Fig. 4:**
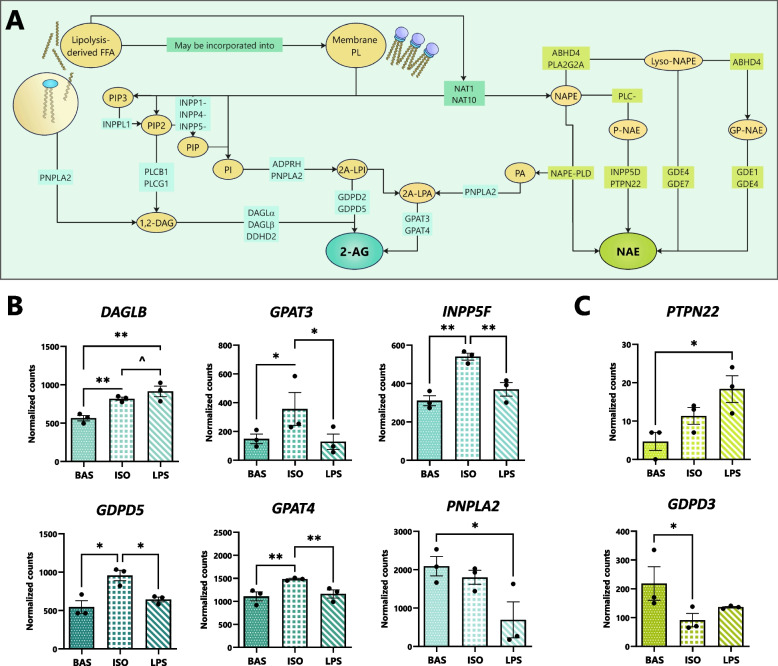
Lipolysis pathways alter the transcription of eCB- and NAE-synthesizing gene networks in bovine adipocytes. Bovine adipocytes were exposed to isoproterenol (ISO; 1 µmol/L), lipopolysaccharide (LPS; 1 µg/mL), or untreated (BAS) for 7 h. **A** Overview of eCB and NAE synthesis pathways and enzymatic catalysts which drive the reactions. **B** and **C** Bar graphs highlighting DESeq2-normalized gene counts associated with 2-AG biosynthesis and NAE biosynthesis in adipocytes. Asterisks denote differences in counts between groups (*P* < 0.1^, < 0.05*, < 0.01**) as determined by Student’s T and Fisher’s LSD. Each dot represents an individual datapoint and error bars, SEM. *n* = 3

To establish the role of canonical and inflammatory lipolysis pathways on the transcriptional regulation of eCB biosynthesis, we evaluated adipocytes’ expression of key gene networks involved (Fig. 4A; reported as DESeq2-normalized gene counts ± SEM with significance set at *P* < 0.05 for differences and *P* < 0.1 for tendencies).

#### 2-AG biosynthesis

As outlined in Fig. 4B, lipolysis pathways differentially regulated the expression of 2-AG-synthesizing genes. Relative to BAS, treatment with ISO or LPS upregulated adipocytes’ transcription of *DAGLB*, and the gene’s expression tended to be greater in LPS than in ISO. Upon exposure to ISO, the expression of several 2-AG synthesizing genes was upregulated versus BAS and LPS, including *GDPD5*, *GPAT3*, *GPAT4*, and *INPP5F.* The presence of LPS reduced adipocytes’ expression of the ATGL-encoding gene, *PNPLA2*, relative to BAS.

#### NAE biosynthesis

Next, we evaluated the expression of NAE-synthesizing enzymes (Fig. 4C). Compared to BAS and ISO, *PTPN22* expression was enhanced by LPS. Exposure to ISO reduced the number of *GDPD3* transcripts relative to BAS.

### Canonical and inflammatory lipolysis differentially regulate the transcription of eCB-degrading enzymes

**Fig. 5:**
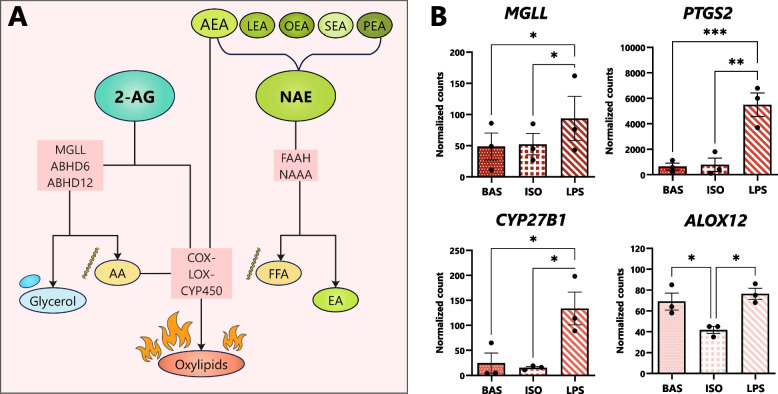
mRNA expression of eCB-degrading enzymes is modulated by lipolysis pathways in dairy cows’ adipocytes. Bovine adipocytes were exposed to isoproterenol (ISO; 1 µmol/L), lipopolysaccharide (LPS; 1 µg/mL), or untreated (BAS) for 7 h. **A** Overview of eCB and NAE degradation pathways depicting hydrolytic and oxidative enzymes involved. **B** Bar graphs depicting DESeq2-normalized gene counts in cows’ adipocytes following 7 h of exposure to BAS, ISO, and LPS. Asterisks denote differences between groups (*P* < 0.05*, < 0.01**, < 0.001***) as determined by Student’s T and Fisher’s LSD of counts; dots represent individual datapoints and error bars, SEM. *n* = 3

Next, we compared the effects of BAS, ISO, and LPS on adipocytes’ expression of oxidizing and hydrolyzing enzymes (Fig. 5). Compared to BAS and ISO, LPS promoted the transcription of the hydrolyzing enzyme *MGLL* and oxidizing *PTGS2* and *CYP27B1.* Expression of *ALOX12* was reduced in ISO relative to BAS and LPS, however, its transcription was comparable amongst the latter treatments.

### Transporters of endocannabinoids and free fatty acids are differentially expressed upon canonical and inflammatory lipolysis

**Fig. 6:**
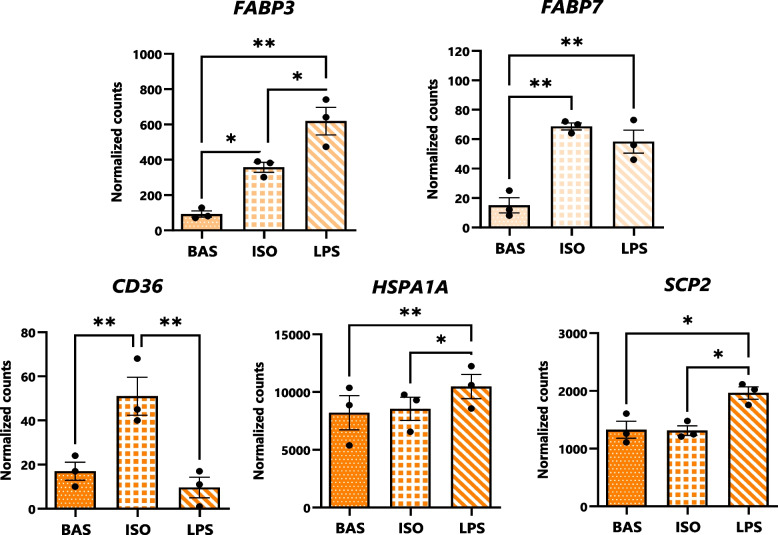
Transcription of FFA-, eCB-, and NAE-transporting genes is altered in dairy cows’ adipocytes upon induction of lipolysis. Bar graphs of DESeq2-normalized gene counts expressed by cultured bovine adipocytes (as determined by bulk RNA-seq analysis) following 7 h of exposure to BAS (untreated), isoproterenol (ISO; 1 µmol/L), and lipopolysaccharide (LPS; 1 µg/mL). Asterisks denote differences between groups (*P* < 0.05*, *P* < 0.01**) as determined by Student’s T and Fisher’s LSD of counts. Dots represent individual datapoints and error bars, SEM. *n* = 3

The expression of genes involved in the transport of FFA, eCBs, and NAEs were then assessed (Fig. 6). Expression of *FABP3* was greater in LPS than in ISO and adipocytes exposed to either treatment exhibited elevated levels over BAS. The transcription of *FABP7* was elevated in ISO and LPS over BAS but its transcription did not differ between ISO and LPS. Adipocytes treated with ISO displayed the greatest transcription of CD36 amongst treatment groups, and counts of the gene did not differ between LPS and BAS. Exposure to LPS enhanced adipocytes’ expression of *HSP1A1* and *SCP2* compared to BAS and ISO.

### ECS receptors, ion channels, and transcription factors are differentially expressed upon stimulation of lipolysis

**Fig. 7:**
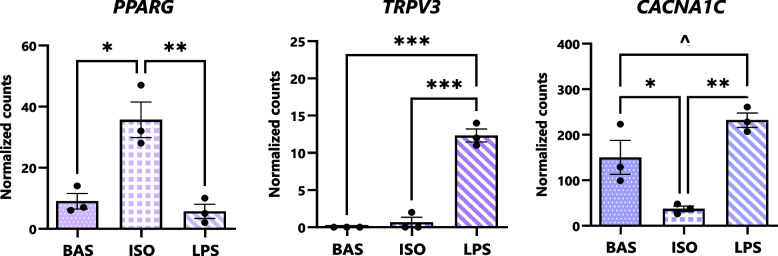
Expression of eCB- and NAE-interacting receptors, ion channels, and transcription factors differ upon stimulation of canonical and inflammatory lipolysis pathways. Bar graphs highlighting DESeq2-normalized gene counts expressed by bovine adipocytes following exposure to isoproterenol (ISO; 1 µmol/L), lipopolysaccharide (LPS; 1 µg/mL), or untreated (BAS) for 7 h. Asterisks denote differences between groups (*P* < 0.1^, < 0.05*, < 0.01**, < 0.001***) as determined by Student’s T and Fisher’s LSD of counts. Dots represent individual datapoints and error bars, SEM. *n* = 3

Next, we assessed transcriptional differences among gene networks associated with the receptors, ion channels, and transcription factors with which eCBs interact (Fig. 7). Exposure to ISO enhanced adipocytes’ expression of *PPARG* relative to BAS and LPS. Expression of *TRPV3* was elevated in LPS relative to BAS and ISO. Transcription of the calcium ion channel-encoding gene, *CACNA1C*, was downregulated in ISO relative to BAS, and its expression tended to be higher in LPS than BAS.

## Discussion

In mammals, the ECS is a potent modulator of metabolic and inflammatory processes which is widely distributed throughout organ systems, including the AT of dairy cows [13, 16, 18]. While drastic shifts in eCB and NAE concentrations and ECS component expression occur within the AT and plasma of PP dairy cows, the factors which drive these changes and their physiologic implications remain largely unknown [13, 18, 49]. Previous studies in rodents and humans have revealed that eCBs are produced within AT and modulate adipocyte FFA mobilization by way of ECS receptor signaling [50]. The present study demonstrates for the first time, however, that lipolysis pathways selectively enhance adipocytes’ production of specific eCBs, which may have significant implications for metabolic and inflammatory responses in PP dairy cows. Through lipidomic and transcriptomic analyses, we identified several enriched pathways and connections between lipolysis pathways and eCB biosynthesis, degradation, transport, and ECS signaling in dairy cows’ adipocytes.

### Stimulation of lipolysis pathways enhances arachidonic acid-based endocannabinoid production by adipocytes

Our data confirmed that, in bovine adipocytes, lipolysis upregulates the overall production of eCBs and NAEs however, the abundances and profiles of each are dependent upon which lipolysis pathway is activated (Fig. 2) [11, 18]. Previous studies have demonstrated strong associations between eCB and NAE concentrations and the abundance of FFA substrates in the diet and plasma, suggesting that precursor availability may play a role in their biosynthesis [51–53]. However, the role of lipolysis on eCB and NAE production had not been previously explored. Our data reveals a link exists between stimulation of lipolysis and the biosynthesis and release of 2-AG and AEA by adipocytes.

Our results indicate that adipocytes’ production of 2-AG is augmented following the stimulation of canonical but not inflammatory lipolysis (Fig. 2). Given the compound’s known involvement in the upregulation of feed intake, nutrient utilization, and regulation of lipolysis, enhanced levels of 2-AG may serve as a mechanism to counter negative energy balance and AT inflammation in near-calving cows, although much remains unknown in this regard [13, 54]. In contrast, our data demonstrate that the biosynthesis and release of AEA are enhanced following inflammatory—but not canonical—lipolysis. Previous studies established that immune cells biosynthesize and secrete AEA in response to LPS; however, the present study’s results are the first to evidence that a similar response is observed among adipocyte populations [55, 56]. These observations align with findings in human macrophages by Melis et al., who not only reported a similar increase in the release of AEA upon LPS exposure, but also proposed that, in additional to availability of precursor FFA (LA and AA), the production of AEA relies upon the degree of inflammation in the extracellular environment [57]. Taken together, these results suggest that the presence of LPS upregulates AEA production. Importantly, AEA concentrations may play a role in regulating the onset and chronic inflammation in AT [58], warranting further investigation into adipocytes’ capacity to synthesize and secrete the molecule, especially in cows with endotoxemia or septicemia.

As previous studies in human subjects have demonstrated that NAE abundances are reflective of precursor FFA profiles, we anticipated that induction of lipolytic pathways would enhance adipocytes’ production of NAEs [51]. However, the results of our study indicate that, in dairy cows’ adipocytes, production and release of LEA, OEA and PEA differ in a lipolysis pathway-specific manner. While beyond the scope of the present study, several conditions may alter the availability of FFA precursors for the biosynthesis of eCBs and NAEs in cows’ adipocytes and should be further explored. For example, in the AT of PP cows undergoing extensive remodeling or inflammation, adipocytes contain altered glycerolipid, glycerophospholipid, glycosphingolipid, and sterol lipid profiles whose acyl chains may serve as substrates for eCB and NAE synthesis [3, 59, 60]. In addition, lipid contents vary widely between differentiating and mature adipocytes and amongst AT depots in rodent and human models [61]. Adding to the complexity of the ECS landscape in AT, certain FFA may be preferentially incorporated into adipocytes’ membrane PL, thus, altering their availability [62]. While presently unknown, future studies should trace FFA released during lipolysis and evaluate their metabolism into eCBs and NAEs. The present findings underscore the complexity of networks which govern eCB and NAE production and release and emphasize the need to characterize the involvement of additional regulatory mechanisms, such as transcription of key genes, in addition to post-translational modifications, which may account for altered activities of these enzymes.

### Canonical and inflammatory lipolysis pathways differentially upregulate the transcription of endocannabinoid synthesizing networks in adipocytes

The transcriptional patterns observed in adipocytes provide novel insight on how canonical and inflammatory lipolysis pathways regulate eCB and NAE profiles in bovine AT (Fig. 4). Corresponding with upregulations in 2-AG release, our data indicates that canonical lipolysis upregulates adipocytes’ expression of key genes associated with the biosynthesis of 2-AG, including *GDPD5* and *GPAT4*, which convert lysophospholipids (2-acyl lysophosphatidic acid and 2-acyl lysophosphatidylinositol) directly into 2-AG, and *INPP5F,* which cleaves phosphatidylinositol 4,5-bisphosphate to free phosphatidylinositol, an intermediate substrate, within the cell membrane [63, 64]. Interestingly, expression of the 2-AG biosynthesizing (and key lipase-encoding) enzyme, *PNPLA2*, was downregulated upon LPS exposure, suggesting bovine adipocytes may exhibit a reduced capacity to synthesize the metabolically active compound 2-AG during endotoxemia.

The results of our present experiments indicate that, in bovine adipocytes, the production of AEA is upregulated in LPS (Fig. 4). At the transcriptional level, adipocytes’ expression of *PTPN22*, which cleaves phospho-AEA to AEA, is upregulated upon exposure to LPS [55]. The distinct shifts in AA-derived eCB release coupled with correspondent changes in transcription presently reported may suggest that, during inflammatory lipolysis, AA is preferentially diverted toward AEA and away from 2-AG biosynthesis. In rodent models, AEA limits TLR-4-mediated release of inflammatory cytokines by immune cells, including TNFα, nitric oxide, IL-1β, and prostaglandins, highlighting the molecule’s capacity to attenuate inflammation [65–67]. While AEA’s anti-inflammatory effects are primarily attributed to its binding and activation of CB2R, its biosynthesis may, in addition, directly limit the amount of free AA available for conversion into pro-inflammatory mediators [8, 68]. Such effects may be particularly beneficial in cows challenged with endotoxemia, wherein AT inflammation may otherwise contribute to dysregulation of lipolysis and predispose cows to disease in subsequent lactations [69, 70].

### Lipolysis pathways modify endocannabinoid-degrading enzyme expression and inflammatory markers in adipocytes

Under inflammatory conditions, eCBs may be rapidly oxidized by COX, LOX, CYP450, or hydrolyzed by MGLL in AT. Importantly, byproducts of eCB oxidation include the bioactive lipid-based mediators of inflammation known as oxylipids, which are associated with increased disease risk in dairy cows [71]. While the affinities of specific enzymes for eCB substrates remain unknown, COX have been shown to display high affinities for AA-based substances [68]. The present results indicate that, when adipocytes are exposed to LPS, their transcription of *PTGS2* (COX-2) and *CYP27B1* are elevated, despite 2-AG levels remaining comparable to basal conditions and AEA release being enhanced (Fig. 5). While the degradation of eCBs may result in the production of pro-inflammatory compounds, the direct implications of lipolysis pathway activation on eCB degradation and oxylipid biosynthesis remain undefined and should be further evaluated through silencing of degradative genes and targeted lipidomic analyses. Additionally, the results of our study indicate that canonical lipolysis does not upregulate adipocytes’ transcription of NAE-degrading enzymes. Collectively, these findings suggest that lower NAE abundances in adipocytes’ extracellular environments during canonical lipolysis may be due to reductions in the compounds’ biosynthesis or transport under these conditions rather than their oxidation or hydrolysis.

### Canonical and inflammatory lipolysis regulate the expression of free fatty acid and endocannabinoid transporters

During lipolysis in PP cows’ AT, FFA are preferentially exported out of the cell, into circulating blood, and partitioned to the mammary gland for milk synthesis [3, 72]. This process is facilitated by the activity of FFA transporters, which may also bind and transport eCBs and NAEs. Known transporters of both FFA and eCBs include fatty acid translocase (CD36), fatty acid-binding proteins (FABP3 and FABP7), heat-shock proteins (Hsp-90), solute carriers (SLC27), and sterol carrier protein-2 (SCP2) [73–75]. Binding of FFA precursors to carrier proteins may alter their availability for eCB or NAE biosynthesis, and the susceptibility of the compounds to oxidative or hydrolytic degradation. The present study revealed that, during canonical and inflammatory lipolysis, adipocytes’ transcription of *FABP3* and *FABP7*, which are associated with the intracellular transport of lipids, are enhanced (Fig. 6). Upon adrenergic stimulation, adipocytes’ transcription of *CD36* is upregulated, corresponding with heightened release of 2-AG. Coinciding with elevations in AEA abundance; however, inflammatory lipolysis upregulates the transcription of *HSP1A1* and *SCP2*, providing further mechanistic evidence for the connection between AT inflammation and adipocytes’ release of AEA. At present, the specificities of certain FFA, eCB, and NAE transporters for various substrates are unknown, however, these proteins may play key roles in the modulation of AT ECS activation and should be further investigated in future studies.

### Canonical and inflammatory lipolysis pathways differentially modulate the expression of endocannabinoid system receptors in bovine adipocytes

In the present study, adipocytes’ expression of *PPARG*—known as the master regulator of adipogenesis—was elevated during canonical, but not inflammatory, lipolysis (Fig. 7). This finding underscores that, within the AT of otherwise healthy cows in negative energy balance, excessive FFA mobilization may be offset through the upregulation of adipogenesis and structural remodeling of AT [3]. In contrast, the AT of transition cows challenged with bacterial infections may exhibit a reduced capacity for adipogenesis and lipogenesis, contributing to the ectopic accumulation of FFA in AT, the blood, and the liver and progression of metabolic diseases in these animals [76]. Furthermore, our data revealed that LPS upregulates the transcription of the transient receptor potential channel, *TRPV3*, in adipocytes. This receptor, whose stimulation is associated with Ca^2+^ influx and suppression of adipogenesis, may contribute to dysregulated lipolysis and inflammation—particularly in the AT of cows afflicted with endotoxemia or septicemia [77]. These observations, largely centered around the regulation of adipogenesis, emphasize ECS receptors’ potential as therapeutic targets for PP cow health and frame the need to advance knowledge in this area.

### Limitations and additional considerations

While the present study elucidated the roles of lipolysis pathways in adipocytes’ biosynthesis and release of eCBs, this model does not mimic the AT environment in vivo. Several AT components–such as nerve fibers, immune cells, and non-adipocyte-derived hormones (e.g., insulin and norepinephrine)–may influence ECS signaling, adipocyte lipolysis, and eCB concentrations in cows’ AT [13]. For example, CB1R activation on peripheral sympathetic nerves has been shown to inhibit their release of norepinephrine, which may modulate the intensity of lipolysis exhibited by adipocytes [78]. In addition, treatment of adipocytes with ISO, a pan-β-AR agonist, does not permit the isolation of effects of specific β-AR isoforms on eCB production. Therefore, to fully elucidate the involvement of β-AR in eCB synthesis and transcriptional regulation in adipocytes, future studies should consider targeted activation of β1-, β2-, and β3-AR with selective agonists. On the other hand, LPS may regulate eCB concentrations and ECS signaling within AT independent of lipolysis. For instance, exposure of AT resident immune cells (such as macrophages) to LPS is known to modulate their secretion of eCBs [79]. While beyond the scope of the present study, the complex interplay between adipocytes, other cell types, and inflammation within cows’ AT on the modulation of ECS signaling warrants further investigation.

## Conclusions

The results of the present study indicate that lipolysis modulates eCB production and release by dairy cows’ adipocytes. Importantly, the profile of eCBs and NAEs released by adipocytes differs in a lipolysis pathway-dependent manner, with canonical lipolysis enhancing 2-AG but reducing LEA and PEA production, and inflammatory lipolysis promoting AEA biosynthesis. Collectively, our findings suggest that negative energy balance (e.g., the PP period, heat stress) and inflammatory conditions (e.g., endotoxemia, septicemia) may alter adipocytes’ transcription of genes associated with eCB and NAE biosynthesis, degradation, transport, and receptor interactions. The regulation of eCB production and ECS signaling by adipocytes may serve as an intrinsic mechanism to counter intense lipolysis and inflammation, holding potential implications for the health and productivity of dairy cows.

### Supplementary Information

**Additional file 1: Supplementary Table 1.** Summary of bulk RNA-seq read counts in bovine adipocytes.

**Additional file 2: Supplementary Fig. 1.** Kyoto Encyclopedia of Genes and Genomes (KEGG) analysis used to compare the transcriptomic profiles of bovine adipocytes following bulk RNA-seq analysis.

**Additional file 3: Supplementary Table 2.** Endocannabinoid system-associated genes of interest assessed in bulk RNA-seq analysis.

## Data Availability

Datasets generated by this study are available upon reasonable request from the corresponding author. Bulk RNA-seq analysis results have been uploaded to the NCBI Gene Expression Omnibus (*upload pending).

## Abbreviations

2-AG: 2-Arachidonoylglycerol
AA: Arachidonic acid
ABHD4: α/β Hydrolase domain-containing 4, *N*-acyl phospholipase B
ABHD5: α/β Hydrolase domain-containing 5, lysophosphatidic acid acyltransferase
ABHD6: α/β Hydrolase domain-containing 6, acylglycerol lipase
AEA: *N*-Arachidonylethanolamide, Anandamide
ALOX12: Arachidonate 12-lipoxygenase, 12S type
ATGL (*PNPLA2*): Adipose triglyceride lipase
β-AR: β-Adrenergic receptor
BSA: Bovine serum albumin
CACNA1C: Voltage-gated T-type calcium channel subunit alpha 1C
CB1R (*CNR1*): Cannabinoid-1 receptor
CB2R (*CNR2*): Cannabinoid-2 receptor
CD36: Fatty acid translocase, scavenger receptor class B member 3
COX: Cyclooxygenase
COX-2 (*PTGS2*): Prostaglandin-endoperoxide synthase 2
CYP: Cytochrome p450 monooxygenase
CYP27B1: Cytochrome P450 monooxygenase family 27 subfamily B member 1
DAG: Diacylglycerol
DAGLα/β: Diacylglycerol lipase alpha/beta
DEG: Differentially expressed gene
ECS: Endocannabinoid system
eCB: Endocannabinoid
ERK1/2: Extracellular signal-regulated protein kinases 1 and 2
FAAH: Fatty acid amide hydrolase
FABP3/7: Fatty acid-binding protein 3/7
FC: Fold-change
FFA: Free fatty acid
GDPD3/5: Glycerophosphodiester phosphodiesterase domain-containing protein 3/5
GPAT: Glycerol-3-phosphate acyltransferase
GPR18/55/119: G protein-coupled receptor 18/55/119
HSL: Hormone-sensitive lipase
Hsp: Heat-shock protein
HspA1A: Heat-shock protein family A member 1A
IL-1β: Interleukin-1 beta
INPP5: Inositol polyphosphate-5-phosphatase
ISO: Isoproterenol
KRBB: Krebs-Ringer bicarbonate buffer
LEA: Linolenoylethanolamide
LOX: Lipoxygenase
LPS: Lipopolysaccharide
MAG: Monoacylglycerol
MAPK: Mitogen-activated protein kinase
MGLL: Monoacylglycerol lipase
NAE: *N*-Acylethanolamine
NAPE-PLD (*NAPEPLD*): *N*-Acylphosphatidylethanolamine-specific phospholipase D
NAT: *N*-Acyltransferase
OEA: Oleoylethanolamide
PBS: Phosphate-buffered saline
PCA: Principal component analysis
PEA: Palmitoylethanolamide
PKA: Protein kinase A
PL: Phospholipid
PLC: Phosphoinositide-specific phospholipase C
PLIN: Perilipin
PLPP: Phospholipid phosphatase
PP: Periparturient
PPARγ: Peroxisome proliferator-activated receptor gamma
PTPN22: Protein tyrosine phosphatase non-receptor type 22
RNA-seq: Bulk RNA sequencing analysis
SCP2: Solute carrier protein 2
SEA: Stearoylethanolamide
TAG: Triacylglycerol
TLR-4: Toll-like receptor 4
TNFα: Tumor necrosis factor alpha
TRPV1/3: Transient receptor potential cation channel subfamily V 1/3
UPLC: Ultra performance liquid chromatography
