# Supplementary material for: Lipolysis pathways modulate lipid mediator release and endocannabinoid system signaling in dairy cows’ adipocytes
Source: J Anim Sci Biotechnol. 2024 Aug 3;15:103. doi: 10.1186/s40104-024-01062-z (PMC11297689; doi:10.1186/s40104-024-01062-z)
Supplement: Supplementary file 1 — Additional file 1: Supplementary Table 1. Summary of bulk RNA-seq read counts in bovine adipocytes. [file 40104_2024_1062_MOESM1_ESM.pdf]

Cow	Treatment	Total reads	Mapped reads	% Mapped	Paired reads	% Paired
1	BAS	62,747,386	59,887,262	95.44%	56,325,124	89.76%
1	ISO	40,372,056	38,583,885	95.57%	36,264,546	89.83%
1	LPS	36,589,636	35,068,054	95.84%	33,174,930	90.67%
2	BAS	46,408,992	44,083,776	94.99%	42,113,306	90.74%
2	ISO	45,553,160	43,302,654	95.06%	41,264,472	90.59%
2	LPS	48,030,546	45,694,994	95.14%	43,636,528	90.85%
3	BAS	30,949,342	29,554,828	95.49%	28,237,382	91.24%
3	ISO	37,959,348	36,194,582	95.35%	34,413,332	90.66%
3	LPS	41,693,172	39,834,383	95.54%	38,233,964	91.70%

Supplementary Table 1. Summary of bulk RNA-seq read counts in bovine adipocytes exposed to isoproterenol (ISO; 1 μ*M*), lipopolysaccharide (LPS; 1 μg/mL), and media only (BAS) for 7 hours.
