# Supplementary figures and images for: Lipolysis pathways modulate lipid mediator release and endocannabinoid system signaling in dairy cows’ adipocytes
Source: J Anim Sci Biotechnol. 2024 Aug 3;15:103. doi: 10.1186/s40104-024-01062-z (PMC11297689; doi:10.1186/s40104-024-01062-z)

ISO vs. BAS

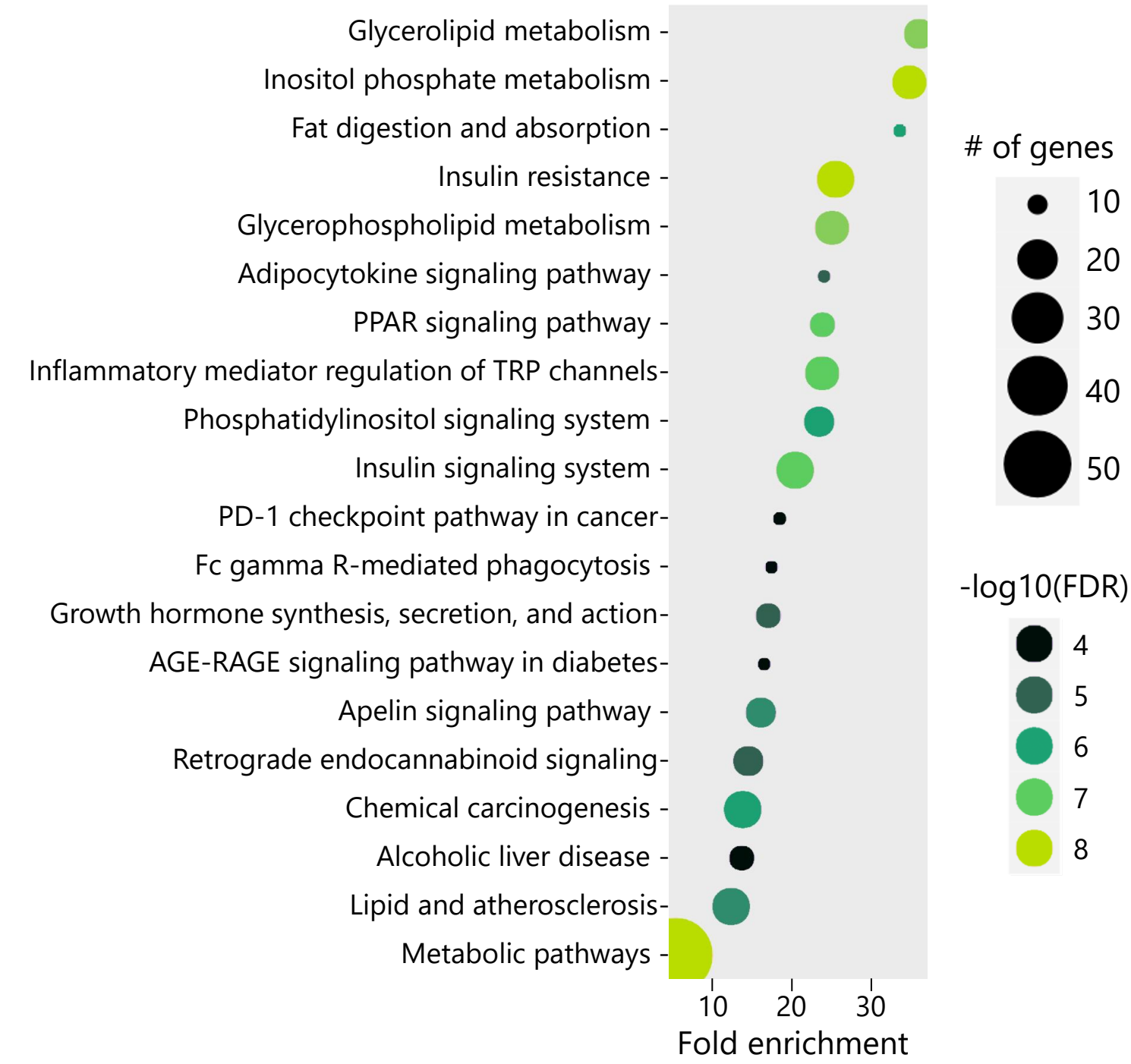

LPS vs. BAS

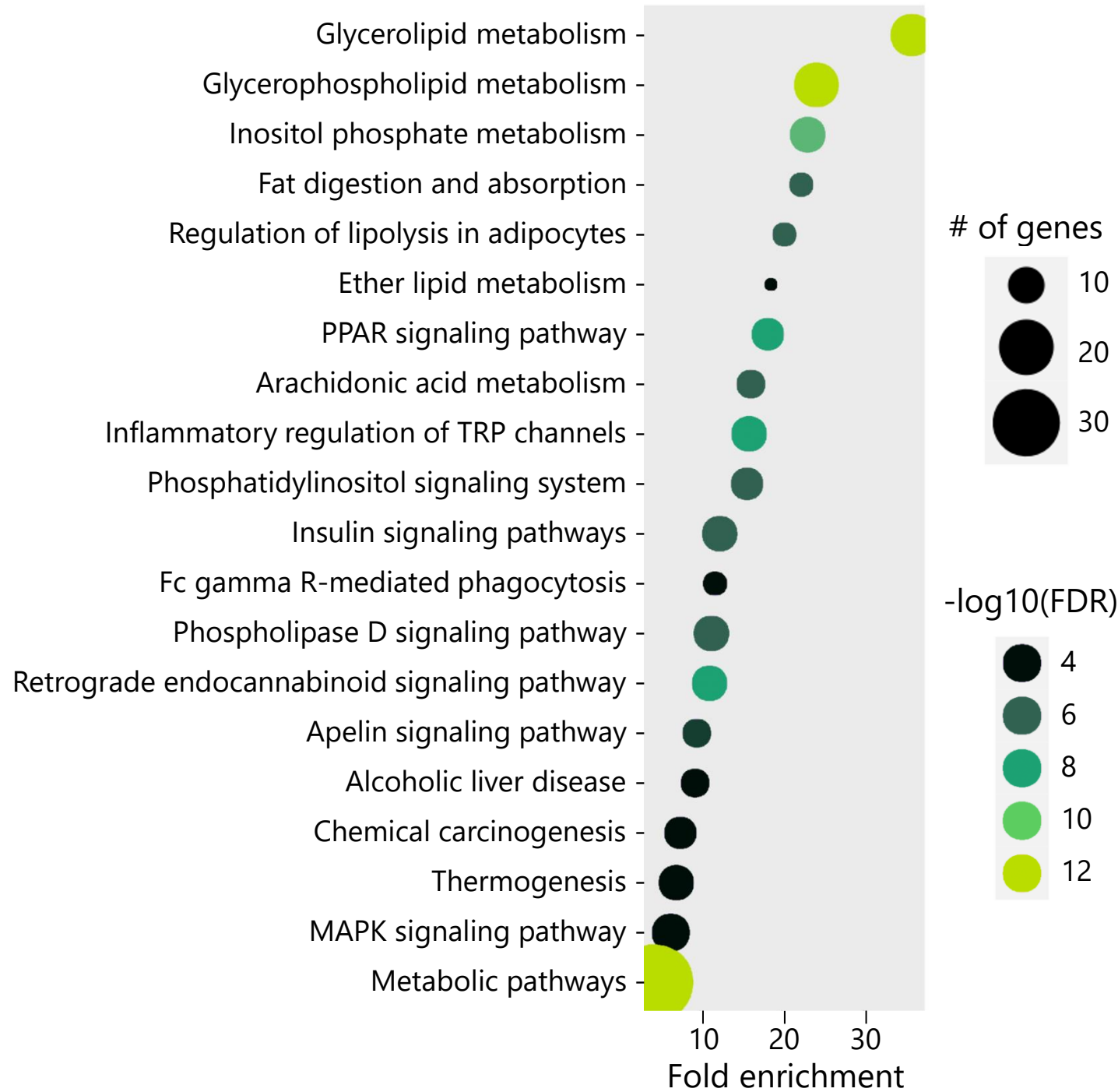

LPS vs. ISO

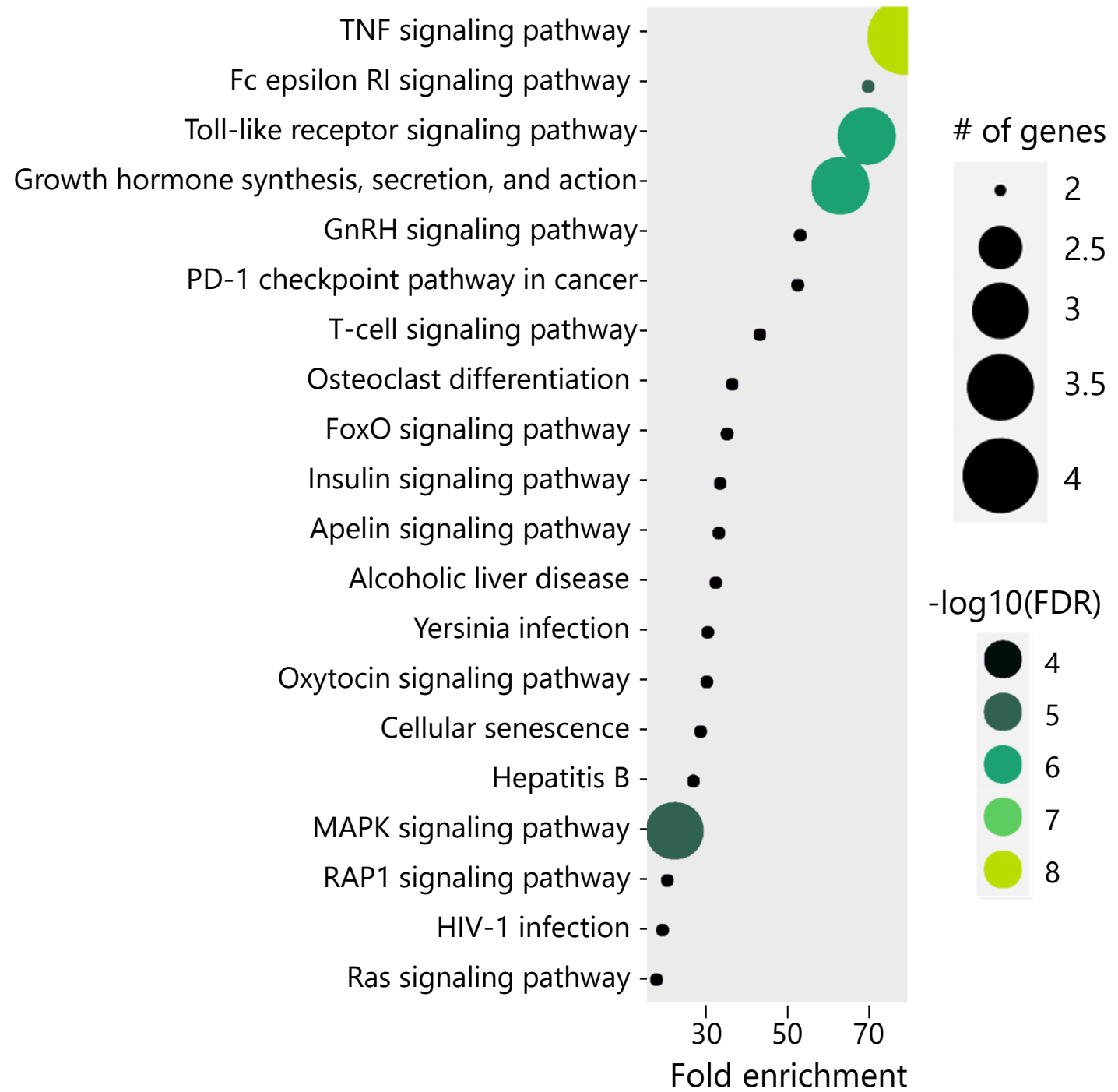

Supplement: Supplementary file 2 — Additional file 2: Supplementary Fig. 1. Kyoto Encyclopedia of Genes and Genomes (KEGG) analysis used to compare the transcriptomic profiles of bovine adipocytes following bulk RNA-seq analysis. [file 40104_2024_1062_MOESM2_ESM.pdf]
