# Supplementary material for: Lipolysis pathways modulate lipid mediator release and endocannabinoid system signaling in dairy cows’ adipocytes
Source: J Anim Sci Biotechnol. 2024 Aug 3;15:103. doi: 10.1186/s40104-024-01062-z (PMC11297689; doi:10.1186/s40104-024-01062-z)
Supplement: Supplementary file 3 — Additional file 3: Supplementary Table 2. Endocannabinoid system-associated genes of interest assessed in bulk RNA-seq analysis. [file 40104_2024_1062_MOESM3_ESM.pdf]

Functional group	Role	Gene abbr.	Ensembl ID	Chr.	Gene start	Gene end	Strand	Length	Description
Biosynthesis of eCB/NAE	Biosynthesis of 2-AG	*ADPRH*	ENSBTAG00000009391	1	64334745	64377235	+	3307	ADP-ribosylarginine hydrolase [Source:VGNC Symbol;Acc:VGNC:25690]
		DAGLA	ENSBTAG00000013942	29	40162375	40188645	+	3126	Bos taurus diacylglycerol lipase alpha (DAGLA), mRNA. [Source:RefSeq mRNA;Acc:NM_001192583]
		DAGLB	ENSBTAG00000009236	25	38297751	38321662	-	4719	Bos taurus diacylglycerol lipase beta (DAGLB), mRNA. [Source:RefSeq mRNA;Acc:NM_001083487]
		DDHD1	ENSBTAG00000019924	10	65673444	65741258	-	7140	DDHD domain containing 1 [Source:VGNC Symbol;Acc:VGNC:27943]
		DDHD2	ENSBTAG00000013145	27	33387283	33416172	+	4307	Bos taurus DDHD domain containing 2 (DDHD2), mRNA. [Source:RefSeq mRNA;Acc:NM_001075598]
		GDPD2	ENSBTAG00000012863	X	80011281	80021117	-	2876	Bos taurus glycerophosphodiester phosphodiesterase domain containing 2 (GDPD2), mRNA. [Source:RefSeq mRNA;Acc:NM_001034471]
		GDPD5	ENSBTAG00000031777	15	54615540	54706765	-	4282	Glycerophosphodiester phosphodiesterase domain containing 5 [Source:VGNC Symbol;Acc:VGNC:29313]
		GPAT3	ENSBTAG00000017592	6	98289617	98360326	+	2465	Bos taurus glycerol-3-phosphate acyltransferase 3 (GPAT3), mRNA. [Source:RefSeq mRNA;Acc:NM_001192514]
		GPAT4	ENSBTAG00000005730	27	36522605	36539773	+	2709	Glycerol-3-phosphate acyltransferase 4 [Source:NCBI gene;Acc:511614]
		INPP4A	ENSBTAG00000004814	11	3650777	3769745	+	8208	Bos taurus inositol polyphosphate-4-phosphatase type IV A (INPP4A), mRNA. [Source:RefSeq mRNA;Acc:NM_001206848]
		INPP4B	ENSBTAG00000014111	17	15374564	15824212	+	3199	Inositol polyphosphate-4-phosphatase type II B [Source:VGNC Symbol;Acc:VGNC:53908]
		PLCB1	ENSBTAG00000008338	13	933264	1794219	+	4694	Bos taurus phospholipase C beta 1 (PLCB1), mRNA. [Source:RefSeq mRNA;Acc:NM_174817]
		PNPLA2	ENSBTAG00000005144	29	50548324	50553197	-	2175	Bos taurus patatin like phospholipase domain containing 2 (PNPLA2), mRNA. [Source:RefSeq mRNA;Acc:NM_001046005]
	Biosynthesis of NAE	*ABHD4*	ENSBTAG00000016658	10	22200351	22212618	-	2364	Bos taurus abhydrolase domain containing 4 (ABHD4), mRNA. [Source:RefSeq mRNA;Acc:NM_001034368]
		GDE1	ENSBTAG00000002101	25	17101520	17127013	-	1638	Bos taurus glycerophosphodiester phosphodiesterase 1 (GDE1), mRNA. [Source:RefSeq mRNA;Acc:NM_001034686]
		GDPD3	ENSBTAG00000016155	25	26186549	26191906	+	1033	Bos taurus glycerophosphodiester phosphodiesterase domain containing 3 (GDPD3), mRNA. [Source:RefSeq mRNA;Acc:NM_001076990]
		NAA10	ENSBTAG00000047702	X	37144907	37149900	-	1225	N(alpha)-acetyltransferase 10, NatA catalytic subunit [Source:NCBI gene;Acc:613636]
		NAA15	ENSBTAG00000004745	X	37144907	37149900	-	1225	N(alpha)-acetyltransferase 10, NatA catalytic subunit [Source:NCBI gene;Acc:613636]

	NAA20	ENSBTAG00000001144	13	39584264	39602876	+	1101	Bos taurus N(alpha)-acetyltransferase 20, NatB catalytic subunit (NAA20), mRNA. [Source:RefSeq mRNA;Acc:NM_001206829]
	NAA25	ENSBTAG00000006666	17	62067802	62129018	+	3112	Bos taurus N(alpha)-acetyltransferase 25, NatB auxiliary subunit (NAA25), mRNA. [Source:RefSeq mRNA;Acc:NM_001206127]
	NAA30	ENSBTAG00000012998	10	69768687	69789526	+	4422	Bos taurus N(alpha)-acetyltransferase 30, NatC catalytic subunit (NAA30), mRNA. [Source:RefSeq mRNA;Acc:NM_001206037]
	NAA35	ENSBTAG00000001016	8	79167865	79255810	+	2956	Bos taurus N(alpha)-acetyltransferase 35, NatC auxiliary subunit (NAA35), mRNA. [Source:RefSeq mRNA;Acc:NM_001193111]
	NAA40	ENSBTAG00000003073	29	42281442	42295820	+	3393	Bos taurus N(alpha)-acetyltransferase 40, NatD catalytic subunit (NAA40), mRNA. [Source:RefSeq mRNA;Acc:NM_001099004]
	NAA50	ENSBTAG00000007784	1	58402278	58433748	-	2942	Bos taurus N(alpha)-acetyltransferase 50, NatE catalytic subunit (NAA50), mRNA. [Source:RefSeq mRNA;Acc:NM_001075750]
	NAA60	ENSBTAG00000004875	25	2859806	2888334	+	3333	Bos taurus N(alpha)-acetyltransferase 60, NatF catalytic subunit (NAA60), mRNA. [Source:RefSeq mRNA;Acc:NM_001075649]
	NAPEPLD	ENSBTAG00000014171	4	44427825	44517650	-	2635	N-acyl phosphatidylethanolamine phospholipase D [Source:VGNC Symbol;Acc:VGNC:31883]
	NAT1	ENSBTAG00000016473	27	39013913	39020598	-	2741	Bos taurus N-acetyltransferase 1 (arylamine N-acetyltransferase) (NAT1), mRNA. [Source:RefSeq mRNA;Acc:NM_001075572]
	NAT10	ENSBTAG00000016747	15	64702013	64740844	+	3843	Bos taurus N-acetyltransferase 10 (NAT10), mRNA. [Source:RefSeq mRNA;Acc:NM_001098031]
	NAT14	ENSBTAG00000019051	18	62021731	62025035	-	2118	N-acetyltransferase 14 (putative) [Source:NCBI gene;Acc:532809]
	NAT9	ENSBTAG00000053909	19	56648056	56652832	+	2714	Bos taurus N-acetyltransferase 9 (putative) (NAT9), mRNA. [Source:RefSeq mRNA;Acc:NM_001103246]
	PTEN	ENSBTAG00000009498	26	9466271	9564180	+	1722	Bos taurus phosphatase and tensin homolog (PTEN), mRNA. [Source:RefSeq mRNA;Acc:NM_001319898]
	PTPN1	ENSBTAG00000006616	13	78534152	78594854	+	1790	Bos taurus protein tyrosine phosphatase, non-receptor type 1 (PTPN1), mRNA. [Source:RefSeq mRNA;Acc:NM_001100326]
	PTPN11	ENSBTAG00000002048	17	61736442	61802172	-	2045	protein tyrosine phosphatase, non-receptor type 11 [Source:VGNC Symbol;Acc:VGNC:33530]
	PTPN12	ENSBTAG00000003825	4	43598679	43690863	-	4088	Bos taurus protein tyrosine phosphatase, non-receptor type 12 (PTPN12), mRNA. [Source:RefSeq mRNA;Acc:NM_001205991]
	PTPN13	ENSBTAG00000014947	6	101531746	101735421	+	6126	protein tyrosine phosphatase, non-receptor type 13 [Source:VGNC Symbol;Acc:VGNC:53774]
	PTPN14	ENSBTAG00000021553	16	69034474	69223706	+	5402	protein tyrosine phosphatase, non-receptor type 14 [Source:VGNC Symbol;Acc:VGNC:33532]
	PTPN18	ENSBTAG00000011658	2	1322993	1352673	+	1527	protein tyrosine phosphatase, non-receptor type 18 [Source:VGNC Symbol;Acc:VGNC:33533]

	PTPN2	ENSBTAG00000010563	24	43097074	43165140	-	1652	Bos taurus protein tyrosine phosphatase, non-receptor type 2 (PTPN2), mRNA. [Source:RefSeq mRNA;Acc:NM_001035431]
	PTPN21	ENSBTAG00000018596	10	100170718	100247414	-	3670	protein tyrosine phosphatase, non-receptor type 21 [Source:VGNC Symbol;Acc:VGNC:33535]
	PTPN22	ENSBTAG00000019617	3	29519988	29575960	+	2427	Bos taurus protein tyrosine phosphatase, non-receptor type 22 (PTPN22), mRNA. [Source:RefSeq mRNA;Acc:NM_001192503]
	PTPN23	ENSBTAG00000002774	22	52263501	52284441	-	5121	Bos taurus protein tyrosine phosphatase, non-receptor type 23 (PTPN23), mRNA. [Source:RefSeq mRNA;Acc:NM_001206526]
	PTPN3	ENSBTAG00000018841	8	98927206	99092707	-	4662	protein tyrosine phosphatase, non-receptor type 3 [Source:VGNC Symbol;Acc:VGNC:33538]
	PTPN4	ENSBTAG00000020855	2	71546908	71733059	+	6553	protein tyrosine phosphatase, non-receptor type 4 [Source:VGNC Symbol;Acc:VGNC:33539]
	PTPN5	ENSBTAG00000020257	29	25991833	26050347	+	3185	Bos taurus protein tyrosine phosphatase, non-receptor type 5 (PTPN5), mRNA. [Source:RefSeq mRNA;Acc:NM_001102293]
	PTPN6	ENSBTAG00000020294	5	103502498	103518817	-	3079	Bos taurus protein tyrosine phosphatase, non-receptor type 6 (PTPN6), mRNA. [Source:RefSeq mRNA;Acc:NM_001098017]
	PTPN7	ENSBTAG00000003016	16	80420337	80428748	+	2986	protein tyrosine phosphatase, non-receptor type 7 [Source:VGNC Symbol;Acc:VGNC:33542]
	PTPN9	ENSBTAG00000016984	21	33305768	33375889	+	3941	Bos taurus protein tyrosine phosphatase, non-receptor type 9 (PTPN9), mRNA. [Source:RefSeq mRNA;Acc:NM_001192054]
Incorp. of FFA into PL membrane	*MBOAT1*	ENSBTAG00000016519	23	37723596	37834307	+	3051	membrane bound O-acyltransferase domain containing 1 [Source:VGNC Symbol;Acc:VGNC:31286]
	MBOAT2	ENSBTAG00000008160	11	88363056	88464714	+	1703	membrane bound O-acyltransferase domain containing 2 [Source:VGNC Symbol;Acc:VGNC:31287]
	MBOAT7	ENSBTAG00000015908	18	63213542	63227739	+	2524	Bos taurus membrane bound O-acyltransferase domain containing 7 (MBOAT7), mRNA. [Source:RefSeq mRNA;Acc:NM_001075152]
Organization of PL membrane, liberation of FFA from PL	*INPP5A*	ENSBTAG00000009181	26	50937626	51083088	-	4973	inositol polyphosphate-5-phosphatase A [Source:VGNC Symbol;Acc:VGNC:30210]
	INPP5D	ENSBTAG00000020173	3	112794123	112931963	+	5495	Bos taurus inositol polyphosphate-5-phosphatase D (INPP5D), mRNA. [Source:RefSeq mRNA;Acc:NM_001101882]
	INPP5E	ENSBTAG00000001354	11	103876330	103886130	-	2950	inositol polyphosphate-5-phosphatase E [Source:VGNC Symbol;Acc:VGNC:30213]
	INPP5F	ENSBTAG00000021801	26	39852025	39946261	+	4727	inositol polyphosphate-5-phosphatase F [Source:NCBI gene;Acc:539889]
	INPP5J	ENSBTAG00000017023	17	69972392	69984070	+	4788	inositol polyphosphate-5-phosphatase J [Source:VGNC Symbol;Acc:VGNC:53909]
	INPP5K	ENSBTAG00000011479	19	22622695	22641597	-	2618	Bos taurus inositol polyphosphate-5-phosphatase K (INPP5K), mRNA. [Source:RefSeq mRNA;Acc:NM_001101986]

	LPCAT1	ENSBTAG00000000944	20	71006062	71048066	+	3512	Bos taurus lysophosphatidylcholine acyltransferase 1 (LPCAT1), mRNA. [Source:RefSeq mRNA;Acc:NM_001206811]
	LPCAT2	ENSBTAG00000019272	18	23786207	23854907	+	2584	lysophosphatidylcholine acyltransferase 2 [Source:VGNC Symbol;Acc:VGNC:30962]
	LPCAT3	ENSBTAG00000013127	5	103451002	103487994	+	2849	Bos taurus lysophosphatidylcholine acyltransferase 3 (LPCAT3), mRNA. [Source:RefSeq mRNA;Acc:NM_001024546]
	LPCAT4	ENSBTAG00000020040	10	28278976	28287059	+	3100	lysophosphatidylcholine acyltransferase 4 [Source:VGNC Symbol;Acc:VGNC:30964]
	LYPD3	ENSBTAG00000018077	18	51674245	51678673	-	2014	Bos taurus LY6/PLAUR domain containing 3 (LYPD3), mRNA. [Source:RefSeq mRNA;Acc:NM_001083419]
	LYPLA2	ENSBTAG00000011625	2	129115781	129121108	-	2585	lysophospholipase II [Source:VGNC Symbol;Acc:VGNC:31106]
	PITPNA	ENSBTAG00000011480	19	22646310	22678320	-	959	phosphatidylinositol transfer protein alpha [Source:VGNC Symbol;Acc:VGNC:32919]
	PITPNB	ENSBTAG00000017799	17	67268876	67330126	-	2980	Bos taurus phosphatidylinositol transfer protein beta (PITPNB), mRNA. [Source:RefSeq mRNA;Acc:NM_001078040]
	PITPNM1	ENSBTAG00000010482	29	45373821	45388348	-	5151	phosphatidylinositol transfer protein membrane associated 1 [Source:VGNC Symbol;Acc:VGNC:32921]
	PITPNM2	ENSBTAG00000001597	17	52428786	52553696	+	9007	Bos taurus phosphatidylinositol transfer protein membrane associated 2 (PITPNM2), mRNA. [Source:RefSeq mRNA;Acc:NM_001191383]
	PITPNM3	ENSBTAG00000002982	19	25321703	25411010	+	5993	PITPNM family member 3 [Source:VGNC Symbol;Acc:VGNC:52807]
	PLA2G10	ENSBTAG00000021522	25	13581741	13601430	-	480	phospholipase A2 group X [Source:HGNC Symbol;Acc:HGNC:9029]
	PLA2G15	ENSBTAG00000007512	18	35623806	35638385	+	3512	phospholipase A2 group XV [Source:VGNC Symbol;Acc:VGNC:32956]
	PLA2G16	ENSBTAG00000015248	29	41963966	41994238	-	761	phospholipase A2, group XVI [Source:NCBI gene;Acc:506802]
	PLA2G1B	ENSBTAG00000026732	17	62647846	62654912	-	623	Bos taurus phospholipase A2 group IB (PLA2G1B), mRNA. [Source:RefSeq mRNA;Acc:NM_174646]
	PLA2G2A	ENSBTAG00000002700	2	132710095	132713984	+	819	Bos taurus phospholipase A2, group IIA (platelets, synovial fluid) (PLA2G2A), mRNA. [Source:RefSeq mRNA;Acc:NM_001025324]
	PLA2G3	ENSBTAG00000011962	17	69982739	69988677	-	2606	Bos taurus phospholipase A2 group III (PLA2G3), mRNA. [Source:RefSeq mRNA;Acc:NM_001080910]
	PLA2G4A	ENSBTAG00000013298	16	67906979	68081283	+	3379	Bos taurus phospholipase A2 group IVA (PLA2G4A), mRNA. [Source:RefSeq mRNA;Acc:NM_001075864]
	PLA2G4B	ENSBTAG00000006784	10	37229548	37241029	+	4945	Bos taurus phospholipase A2, group IVB (cytosolic) (PLA2G4B), mRNA. [Source:RefSeq mRNA;Acc:NM_001114657]
	PLA2G4F	ENSBTAG00000032068	10	37533181	37547704	-	3070	Bos taurus phospholipase A2 group IVF (PLA2G4F), mRNA. [Source:RefSeq mRNA;Acc:NM_001192569]

		PLA2G5	ENSBTAG00000039122	2	132630420	132665213	-	2690	Bos taurus phospholipase A2 group V (PLA2G5), mRNA. [Source:RefSeq mRNA;Acc:NM_001193052]
		PLA2G6	ENSBTAG00000014015	5	109891806	109938952	-	3493	phospholipase A2 group VI [Source:VGNC Symbol;Acc:VGNC:32964]
		PLA2G7	ENSBTAG00000019315	23	19924777	19963342	-	2985	phospholipase A2 group VII [Source:VGNC Symbol;Acc:VGNC:32965]
		PLCB4	ENSBTAG00000013116	13	2304288	2503503	+	5044	Bos taurus phospholipase C beta 4 (PLCB4), mRNA. [Source:RefSeq mRNA;Acc:NM_001166510]
		PLCD1	ENSBTAG00000037726	22	11459788	11481247	-	2784	Bos taurus phospholipase C delta 1 (PLCD1), mRNA. [Source:RefSeq mRNA;Acc:NM_001081576]
		PLCD3	ENSBTAG00000006052	19	44781286	44802191	-	2983	Bos taurus phospholipase C delta 3 (PLCD3), mRNA. [Source:RefSeq mRNA;Acc:NM_001193028]
		PLCD4	ENSBTAG00000003809	2	106575924	106598631	+	3220	Bos taurus phospholipase C delta 4 (PLCD4), mRNA. [Source:RefSeq mRNA;Acc:NM_001046489]
		PLCG1	ENSBTAG00000017584	13	69791010	69824050	+	5451	phospholipase C gamma 1 [Source:VGNC Symbol;Acc:VGNC:32988]
		PLCG2	ENSBTAG00000002103	18	8314287	8467665	+	3858	phospholipase C gamma 2 [Source:VGNC Symbol;Acc:VGNC:32989]
		PLCH1	ENSBTAG00000008307	1	111945804	112167879	+	5113	phospholipase C eta 1 [Source:VGNC Symbol;Acc:VGNC:32990]
		PLCH2	ENSBTAG00000002464	16	50361199	50426588	-	5811	phospholipase C eta 2 [Source:VGNC Symbol;Acc:VGNC:32991]
		PLD1	ENSBTAG00000017490	1	95742044	95963408	+	4914	Bos taurus phospholipase D1, phosphatidylcholine-specific (PLD1), mRNA. [Source:RefSeq mRNA;Acc:NM_001102001]
		PLD2	ENSBTAG00000013392	19	26543144	26556510	-	3898	phospholipase D2 [Source:VGNC Symbol;Acc:VGNC:32996]
		PLPP1	ENSBTAG00000010526	20	23636355	23738916	+	1674	Bos taurus phospholipid phosphatase 1 (PLPP1), mRNA. [Source:RefSeq mRNA;Acc:NM_001080329]
		PLPP2	ENSBTAG00000000717	7	43016868	43028554	-	1354	Bos taurus phospholipid phosphatase 2 (PLPP2), mRNA. [Source:RefSeq mRNA;Acc:NM_001045890]
		PLPP3	ENSBTAG00000011640	3	89608083	89696904	+	2753	phospholipid phosphatase 3 [Source:VGNC Symbol;Acc:VGNC:33046]
		PLPP4	ENSBTAG00000032106	26	40509769	40657136	+	2002	phospholipid phosphatase 4 [Source:VGNC Symbol;Acc:VGNC:33047]
		PLPP5	ENSBTAG00000027635	27	33416459	33421456	-	1844	phospholipid phosphatase 5 [Source:VGNC Symbol;Acc:VGNC:33048]
		PLPP6	ENSBTAG00000011050	8	39863787	39864933	-	1147	Bos taurus phospholipid phosphatase 6 (PLPP6), mRNA. [Source:RefSeq mRNA;Acc:NM_001024573]
Degradation of eCB/NAE	Oxidation of eCB	*ALOX12*	ENSBTAG00000021933	19	26787284	26800417	+	2354	Bos taurus arachidonate 12-lipoxygenase, 12S type (ALOX12), mRNA. [Source:RefSeq mRNA;Acc:NM_001192336]
		ALOX12B	ENSBTAG00000012475	19	27718183	27730008	-	2427	arachidonate 12-lipoxygenase, 12R type [Source:VGNC Symbol;Acc:VGNC:25842]
		ALOX12E	ENSBTAG00000031933	19	26727566	26736433	+	2276	Bos taurus arachidonate lipoxygenase, epidermal (ALOX12E), mRNA. [Source:RefSeq mRNA;Acc:NM_001083532]

	ALOX15	ENSBTAG00000011990	19	26697231	26705809	+	2885	Bos taurus arachidonate 15-lipoxygenase (ALOX15), mRNA. [Source:RefSeq mRNA;Acc:NM_174501]
	ALOX5	ENSBTAG00000020319	28	44346285	44392546	-	2655	Bos taurus arachidonate 5-lipoxygenase (ALOX5), mRNA. [Source:RefSeq mRNA;Acc:NM_001192792]
	ALOXE3	ENSBTAG00000012478	19	27739350	27760708	-	3724	arachidonate lipoxygenase 3 [Source:VGNC Symbol;Acc:VGNC:25846]
	CYP11A1	ENSBTAG00000006934	21	34328403	34342900	+	2041	Bos taurus cytochrome P450, family 11, subfamily A, polypeptide 1 (CYP11A1), mRNA. [Source:RefSeq mRNA;Acc:NM_176644]
	CYP17A1	ENSBTAG00000069547	26	23450403	23456668	-	1785	Bos taurus cytochrome P450, family 17, subfamily A, polypeptide 1 (CYP17A1), mRNA. [Source:RefSeq mRNA;Acc:NM_174304]
	CYP1A1	ENSBTAG00000001021	21	33951654	33955241	+	2547	cytochrome P450, subfamily I (aromatic compound-inducible), polypeptide 1 [Source:NCBI gene;Acc:282870]
	CYP1A2	ENSBTAG00000000085	21	33923110	33930074	-	2027	cytochrome P450, family 1, subfamily A, polypeptide 2 [Source:NCBI gene;Acc:503552]
	CYP1B1	ENSBTAG00000010531	11	20470965	20476592	-	2796	Bos taurus cytochrome P450, family 1, subfamily B, polypeptide 1 (CYP1B1), mRNA. [Source:RefSeq mRNA;Acc:NM_001192294]
	CYP20A1	ENSBTAG00000000851	2	91483594	91527751	+	1680	Bos taurus cytochrome P450, family 20, subfamily A, polypeptide 1 (CYP20A1), mRNA. [Source:RefSeq mRNA;Acc:NM_001015644]
	CYP21	ENSBTAG00000047039	23	27326531	27330230	-	2154	Bos taurus cytochrome P450, family 21, subfamily A, polypeptide 2 (CYP21A2), mRNA. [Source:RefSeq mRNA;Acc:NM_174639]
	CYP26B1	ENSBTAG00000012212	11	12376837	12396521	+	5373	cytochrome P450, family 26, subfamily B, polypeptide 1 [Source:NCBI gene;Acc:540868]
	CYP27A1	ENSBTAG00000013489	2	106739389	106782307	+	2436	cytochrome P450 family 27 subfamily A member 1 [Source:HGNC Symbol;Acc:HGNC:2605]
	CYP27B1	ENSBTAG00000016906	5	55698030	55704141	+	3361	cytochrome P450, family 27, subfamily B, polypeptide 1 [Source:NCBI gene;Acc:539630]
	CYP2B6	ENSBTAG00000003871	18	50290687	50308138	-	2607	Bos taurus cytochrome P450 subfamily 2B (CYP2B6), mRNA. [Source:RefSeq mRNA;Acc:NM_001075173]
	CYP2D14	ENSBTAG00000026501	5	113086815	113094984	-	4669	cytochrome P450, family 2, subfamily D, polypeptide 6 [Source:NCBI gene;Acc:282211]
	CYP2R1	ENSBTAG00000026502	15	37889793	37958129	+	3153	Bos taurus cytochrome P450, family 2, subfamily R, polypeptide 1 (CYP2R1), mRNA. [Source:RefSeq mRNA;Acc:NM_001076267]
	CYP2S1	ENSBTAG00000010419	18	50379572	50393561	+	2970	Bos taurus cytochrome P450, family 2, subfamily S, polypeptide 1 (CYP2S1), mRNA. [Source:RefSeq mRNA;Acc:NM_001100366]
	CYP2U1	ENSBTAG00000018240	6	17261763	17282966	-	4589	Bos taurus cytochrome P450, family 2, subfamily U, polypeptide 1 (CYP2U1), mRNA. [Source:RefSeq mRNA;Acc:NM_001076050]
	CYP2W1	ENSBTAG00000012972	25	41748481	41752614	-	1482	cytochrome P450 family 2 subfamily W member 1 [Source:HGNC Symbol;Acc:HGNC:20243]

	CYP39A1	ENSBTAG00000017701	23	19743358	19878413	-	5302	Bos taurus cytochrome P450, family 39, subfamily A, polypeptide 1 (CYP39A1), mRNA. [Source:RefSeq mRNA;Acc:NM_001098938]
	CYP3A5	ENSBTAG00000053645	25	36648051	36691853	-	2002	cytochrome P450, family 3, subfamily A, polypeptide 5 [Source:NCBI gene;Acc:526682]
	CYP46A1	ENSBTAG00000035544	21	64668944	64697472	+	2311	Bos taurus cytochrome P450, family 46, subfamily A, polypeptide 1 (CYP46A1), mRNA. [Source:RefSeq mRNA;Acc:NM_001076810]
	CYP4V2	ENSBTAG00000021263	27	16249747	16266690	+	4766	Bos taurus cytochrome P450, family 4, subfamily V, polypeptide 2 (CYP4V2), mRNA. [Source:RefSeq mRNA;Acc:NM_001034373]
	CYP51A1	ENSBTAG00000001992	4	9459764	9476713	-	2761	Bos taurus cytochrome P450, family 51, subfamily A, polypeptide 1 (CYP51A1), mRNA. [Source:RefSeq mRNA;Acc:NM_001025319]
	CYP7B1	ENSBTAG00000001299	14	29199622	29460150	-	4136	cytochrome P450 family 7 subfamily B member 1 [Source:HGNC Symbol;Acc:HGNC:2652]
	CYP8B1	ENSBTAG00000056462	22	14946435	14949705	-	1055	Bos taurus cytochrome P450, family 8, subfamily B, polypeptide 1 (CYP8B1), mRNA. [Source:RefSeq mRNA;Acc:NM_001076139]
	PTGS1	ENSBTAG00000006716	11	93236273	93262107	+	2786	Bos taurus prostaglandin-endoperoxide synthase 1 (PTGS1), mRNA. [Source:RefSeq mRNA;Acc:NM_001105323]
	PTGS2	ENSBTAG00000014127	16	67728006	67735629	-	3489	Bos taurus prostaglandin-endoperoxide synthase 2 (PTGS2), mRNA. [Source:RefSeq mRNA;Acc:NM_174445]
Hydrolysis of 2-AG	*ABHD1*	ENSBTAG00000007077	11	72504307	72515568	-	5603	abhydrolase domain containing 1 [Source:VGNC Symbol;Acc:VGNC:25487]
	ABHD10	ENSBTAG00000004601	1	56737576	56750712	+	1409	Bos taurus abhydrolase domain containing 10 (ABHD10), mRNA. [Source:RefSeq mRNA;Acc:NM_001015606]
	ABHD11	ENSBTAG00000010339	25	33530965	33533374	+	1259	abhydrolase domain containing 11 [Source:VGNC Symbol;Acc:VGNC:25489]
	ABHD12	ENSBTAG00000001420	13	42813204	42879226	-	3003	Bos taurus abhydrolase domain containing 12 (ABHD12), mRNA. [Source:RefSeq mRNA;Acc:NM_001078116]
	ABHD12B	ENSBTAG00000044049	10	43738868	43756260	+	1065	abhydrolase domain containing 12B [Source:VGNC Symbol;Acc:VGNC:25491]
	ABHD13	ENSBTAG00000067980	12	83604977	83618953	+	1295	Bos taurus abhydrolase domain containing 13 (ABHD13), mRNA. [Source:RefSeq mRNA;Acc:NM_001101089]
	ABHD14A	ENSBTAG00000053854	22	48954795	48957705	-	1251	abhydrolase domain containing 14A [Source:HGNC Symbol;Acc:HGNC:24538]
	ABHD15	ENSBTAG00000025167	19	20816663	20821639	-	1464	Bos taurus abhydrolase domain containing 15 (ABHD15), mRNA. [Source:RefSeq mRNA;Acc:NM_001206398]
	ABHD16B	ENSBTAG00000031906	13	53957078	53958761	-	1684	Bos taurus abhydrolase domain containing 16B (ABHD16B), mRNA. [Source:RefSeq mRNA;Acc:NM_001038541]

		ABHD17B	ENSBTAG00000006816	8	48108615	48157205	-	2505	Bos taurus abhydrolase domain containing 17B (ABHD17B), mRNA. [Source:RefSeq mRNA;Acc:NM_001101278]
		ABHD18	ENSBTAG00000010630	17	29695955	29733214	-	2301	abhydrolase domain containing 18 [Source:VGNC Symbol;Acc:VGNC:25499]
		ABHD2	ENSBTAG00000019954	21	20576003	20684305	+	1968	Bos taurus abhydrolase domain containing 2 (ABHD2), mRNA. [Source:RefSeq mRNA;Acc:NM_001015549]
		ABHD3	ENSBTAG00000005709	24	34541295	34591097	+	2053	Bos taurus abhydrolase domain containing 3 (ABHD3), mRNA. [Source:RefSeq mRNA;Acc:NM_001076187]
		ABHD5	ENSBTAG00000008416	22	15584972	15626415	+	1989	Bos taurus abhydrolase domain containing 5 (ABHD5), mRNA. [Source:RefSeq mRNA;Acc:NM_001076063]
		ABHD6	ENSBTAG00000016615	22	43026804	43075099	-	2096	Bos taurus abhydrolase domain containing 6 (ABHD6), mRNA. [Source:RefSeq mRNA;Acc:NM_001075196]
		MGLL	ENSBTAG00000018248	22	59639050	59826846	+	3927	Bos taurus monoglyceride lipase (MGLL), mRNA. [Source:RefSeq mRNA;Acc:NM_001206681]
	Hydrolysis of NAE	*NAAA*	ENSBTAG00000019478	6	90770495	90794386	-	1244	Bos taurus N-acylethanolamine acid amidase (NAAA), mRNA. [Source:RefSeq mRNA;Acc:NM_001100369]
		FAAH	ENSBTAG00000007507	3	99716645	99737813	-	2470	Bos taurus fatty acid amide hydrolase (FAAH), mRNA. [Source:RefSeq mRNA;Acc:NM_001099102]
eCB/NAE-interacting receptors, transcription factors, ion channels	eCB receptors	*CNR1*	ENSBTAG00000054523	9	61744572	61774118	+	5616	Bos taurus cannabinoid receptor 1 (CNR1), mRNA. [Source:RefSeq mRNA;Acc:NM_001242341]
		CNR2	ENSBTAG00000019371	2	129001537	129038262	+	2489	Bos taurus cannabinoid receptor 2 (CNR2), mRNA. [Source:RefSeq mRNA;Acc:NM_001192303]
	eCB/NAE receptors	*GPR119*	ENSBTAG00000014770	X	14348502	14349509	-	1008	G protein-coupled receptor 119 [Source:VGNC Symbol;Acc:VGNC:29546]
		GPR18	ENSBTAG00000002240	12	76118033	76121780	-	1381	Bos taurus G protein-coupled receptor 18 (GPR18), mRNA. [Source:RefSeq mRNA;Acc:NM_001034689]
		GPR55	ENSBTAG00000059603	2	118714413	118715459	-	1047	G protein-coupled receptor 55 [Source:VGNC Symbol;Acc:VGNC:29591]
	Transcription factors	*PPARA*	ENSBTAG00000008063	5	116438987	116507065	+	2732	peroxisome proliferator activated receptor alpha [Source:VGNC Symbol;Acc:VGNC:33181]
		PPARG	ENSBTAG00000001333	22	56709248	56835386	-	4726	peroxisome proliferator activated receptor gamma [Source:VGNC Symbol;Acc:VGNC:33183]
		PPARGC1A	ENSBTAG00000017024	6	43380463	43501184	-	6584	Bos taurus PPARG coactivator 1 alpha (PPARGC1A), mRNA. [Source:RefSeq mRNA;Acc:NM_177945]
		PPARGC1B	ENSBTAG00000012943	7	61073587	61186881	+	3317	PPARG coactivator 1 beta [Source:VGNC Symbol;Acc:VGNC:33185]
	Ion channels	*CACNA1A*	ENSBTAG00000014828	7	12144237	12396421	+	7499	Bos taurus calcium voltage-gated channel subunit alpha1 A (CACNA1A), mRNA. [Source:RefSeq mRNA;Acc:NM_001075129]
		CACNA1B	ENSBTAG00000007882	11	106722392	106933600	+	8015	Bos taurus calcium voltage-gated channel subunit alpha1 B (CACNA1B), mRNA. [Source:RefSeq mRNA;Acc:NM_174632]
		CACNA1C	ENSBTAG00000010660	5	108446722	108792612	+	7626	calcium voltage-gated channel subunit alpha1 C [Source:HGNC Symbol;Acc:HGNC:1390]

		CACNA1D	ENSBTAG00000010026	22	47162857	47508559	-	6634	Bos taurus calcium voltage-gated channel subunit alpha1 D (CACNA1D), mRNA. [Source:RefSeq mRNA;Acc:NM_001193025]
		CACNA1G	ENSBTAG00000009835	19	36102387	36164028	-	7927	Bos taurus calcium voltage-gated channel subunit alpha1 G (CACNA1G), mRNA. [Source:RefSeq mRNA;Acc:NM_001193140]
		CACNA1H	ENSBTAG00000026461	25	930615	988927	+	7650	calcium voltage-gated channel subunit alpha1 H [Source:VGNC Symbol;Acc:VGNC:26678]
		CACNA1S	ENSBTAG00000047491	16	79599108	79660665	-	7865	calcium voltage-gated channel subunit alpha1 S [Source:HGNC Symbol;Acc:HGNC:1397]
		CACNA2D2	ENSBTAG00000009489	22	49872343	49986489	+	4785	calcium voltage-gated channel auxiliary subunit alpha2delta 2 [Source:VGNC Symbol;Acc:VGNC:26680]
		TRPA1	ENSBTAG00000002062	14	35611321	35666918	-	4222	transient receptor potential cation channel subfamily A member 1 [Source:VGNC Symbol;Acc:VGNC:36380]
		TRPV1	ENSBTAG00000018880	19	24279228	24301269	-	2635	transient receptor potential cation channel subfamily V member 1 [Source:VGNC Symbol;Acc:VGNC:36397]
		TRPV2	ENSBTAG00000003014	19	33213076	33230717	-	2553	Bos taurus transient receptor potential cation channel subfamily V member 2 (TRPV2), mRNA. [Source:RefSeq mRNA;Acc:NM_001024493]
		TRPV3	ENSBTAG00000000020	19	24239065	24273712	-	6684	transient receptor potential cation channel subfamily V member 3 [Source:VGNC Symbol;Acc:VGNC:36399]
		TRPV4	ENSBTAG00000000031	17	63410753	63447169	+	3213	Bos taurus transient receptor potential cation channel subfamily V member 4 (TRPV4), mRNA. [Source:RefSeq mRNA;Acc:NM_001192385]
Transport of FFA, eCB, and NAE	Transporters	*CD36*	ENSBTAG00000017866	4	40380301	40443336	-	4214	Bos taurus CD36 molecule (CD36), transcript variant 1, mRNA. [Source:RefSeq mRNA;Acc:NM_001278621]
		FABP2	ENSBTAG00000017045	6	5970114	5973346	-	709	Bos taurus fatty acid binding protein 2 (FABP2), mRNA. [Source:RefSeq mRNA;Acc:NM_001025332]
		FABP3	ENSBTAG00000016819	2	122285620	122294666	+	1341	fatty acid binding protein 3 [Source:VGNC Symbol;Acc:VGNC:28697]
		FABP4	ENSBTAG00000037526	14	44676542	44681059	-	754	fatty acid binding protein 4 [Source:HGNC Symbol;Acc:HGNC:3559]
		FABP5	ENSBTAG00000047330	14	44488895	44494877	+	1839	Bos taurus fatty acid binding protein 5 (FABP5), mRNA. [Source:RefSeq mRNA;Acc:NM_174315]
		FABP6	ENSBTAG00000010632	7	71800104	71806269	+	615	fatty acid binding protein 6 [Source:VGNC Symbol;Acc:VGNC:28698]
		FABP7	ENSBTAG00000033803	9	28476062	28479847	-	814	Bos taurus fatty acid binding protein 7 (FABP7), mRNA. [Source:RefSeq mRNA;Acc:NM_001078162]
		HSPA1A	ENSBTAG00000025441	23	27520317	27522790	-	2474	Bos taurus heat shock protein family A (Hsp70) member 1A (HSPA1A), mRNA. [Source:RefSeq mRNA;Acc:NM_203322]
		HSPA4	ENSBTAG00000015683	7	44607161	44661126	+	4839	Bos taurus heat shock protein family A (Hsp70) member 4 (HSPA4), mRNA. [Source:RefSeq mRNA;Acc:NM_001114192]
		HSPA5	ENSBTAG00000007662	11	96115141	96119503	-	2541	Bos taurus heat shock protein family A (Hsp70) member 5 (HSPA5), mRNA. [Source:RefSeq mRNA;Acc:NM_001075148]

	HSPA8	ENSBTAG00000013162	15	33679009	33683436	-	2317	Bos taurus heat shock protein family A (Hsp70) member 8 (HSPA8), mRNA. [Source:RefSeq mRNA;Acc:NM_174345]
	HSPA9	ENSBTAG00000011419	7	49894161	49909804	-	2651	heat shock protein family A (Hsp70) member 9 [Source:VGNC Symbol;Acc:VGNC:52243]
	SCP2	ENSBTAG00000003746	3	93275045	93393567	-	2624	Bos taurus sterol carrier protein 2 (SCP2), mRNA. [Source:RefSeq mRNA;Acc:NM_001033990]
	SLC27A1	ENSBTAG00000016775	7	5605375	5646428	-	3634	Bos taurus solute carrier family 27 member 1 (SLC27A1), mRNA. [Source:RefSeq mRNA;Acc:NM_001033625]
	SLC27A3	ENSBTAG00000021862	3	16619248	16624026	-	2433	solute carrier family 27 member 3 [Source:VGNC Symbol;Acc:VGNC:34787]
	SLC27A4	ENSBTAG00000015436	11	98945593	98959912	+	2722	Bos taurus solute carrier family 27 member 4 (SLC27A4), mRNA. [Source:RefSeq mRNA;Acc:NM_001075667]
	SLC27A5	ENSBTAG00000015164	18	65732865	65741718	-	2285	Bos taurus solute carrier family 27 member 5 (SLC27A5), mRNA. [Source:RefSeq mRNA;Acc:NM_001103273]

Supplementary Table 2. Endocannabinoid system-associated genes of interest assessed in bulk RNA-seq analysis of cultured bovine adipocytes exposed to isoproterenol (ISO; 1 μ*M*), lipopolysaccharide (LPS; 1 μg/mL), and media alone (BAS) for 7 hours.
